# Characterization of promoter elements of isoprene‐responsive genes and the ability of isoprene to bind START domain transcription factors

**DOI:** 10.1002/pld3.483

**Published:** 2023-01-31

**Authors:** Sarathi M. Weraduwage, Abira Sahu, Martin Kulke, Josh V. Vermaas, Thomas D. Sharkey

**Affiliations:** ^1^ MSU‐DOE Plant Research Laboratory Michigan State University East Lansing Michigan USA; ^2^ Department of Biochemistry and Molecular Biology Michigan State University East Lansing Michigan USA; ^3^ Great Lakes Bioenergy Research Center Michigan State University East Lansing Michigan USA; ^4^ Plant Resilience Institute Michigan State University East Lansing Michigan USA

**Keywords:** AGRIS, cis‐regulatory elements, class IV HD‐ZIP family START domain transcription factor, membrane‐bound receptor, molecular simulation, protein modeling

## Abstract

Isoprene has recently been proposed to be a signaling molecule that can enhance tolerance of both biotic and abiotic stress. Not all plants make isoprene, but all plants tested to date respond to isoprene. We hypothesized that isoprene interacts with existing signaling pathways rather than requiring novel mechanisms for its effect on plants. We analyzed the cis‐regulatory elements (CREs) in promoters of isoprene‐responsive genes and the corresponding transcription factors binding these promoter elements to obtain clues about the transcription factors and other proteins involved in isoprene signaling. Promoter regions of isoprene‐responsive genes were characterized using the Arabidopsis cis‐regulatory element database. CREs bind ARR1, Dof, DPBF, bHLH112, GATA factors, GT‐1, MYB, and WRKY transcription factors, and light‐responsive elements were overrepresented in promoters of isoprene‐responsive genes; CBF‐, HSF‐, WUS‐binding motifs were underrepresented. Transcription factors corresponding to CREs overrepresented in promoters of isoprene‐responsive genes were mainly those important for stress responses: drought‐, salt/osmotic‐, oxidative‐, herbivory/wounding and pathogen‐stress. More than half of the isoprene‐responsive genes contained at least one binding site for TFs of the class IV (homeodomain leucine zipper) HD‐ZIP family, such as GL2, ATML1, PDF2, HDG11, ATHB17. While the HD‐zipper‐loop‐zipper (ZLZ) domain binds to the L1 box of the promoter region, a special domain called the steroidogenic acute regulatory protein‐related lipid transfer, or START domain, can bind ligands such as fatty acids (e.g., linolenic and linoleic acid). We tested whether isoprene might bind in such a START domain. Molecular simulations and modeling to test interactions between isoprene and a class IV HD‐ZIP family START‐domain‐containing protein were carried out. Without membrane penetration by the HDG11 START domain, isoprene within the lipid bilayer was inaccessible to this domain, preventing protein interactions with membrane bound isoprene. The cross‐talk between isoprene‐mediated signaling and other growth regulator and stress signaling pathways, in terms of common CREs and transcription factors could enhance the stability of the isoprene emission trait when it evolves in a plant but so far it has not been possible to say what how isoprene is sensed to initiate signaling responses.

## 1 INTRODUCTION

Isoprene is a biogenic volatile hemiterpene and recently proposed to be a novel signaling molecule in plants (Dani et al., 2022; Harvey & Sharkey, 2016; Vanzo et al., 2016; Zuo et al., 2019). Isoprene synthase (ISPS) catalyzes the conversion of dimethylallyl diphosphate (DMADP), a product of the chloroplastic methylerythritol 4‐phosphate (MEP) pathway, to isoprene (Affek & Yakir, 2002; Schwender et al., 1997; Sharkey et al., 2001,  2013; Tattini et al., 2014). Isoprene can enhance tolerance of high temperature, drought, O_3_ and oxidative stress, and herbivory in plants (Affek & Yakir, 2002; Behnke et al., 2007, 2010; Laothawornkitkul et al., 2008; Loreto et al., 2001; Sharkey et al., 2001; Singsaas et al., 1997; Tattini et al., 2014; Velikova et al., 2011; Vickers, Possell, et al., 2009). Harvey et al. (2015) showed that the proposed role for isoprene as a membrane stabilizer or an antioxidant molecule (Pollastri et al., 2021; Sharkey et al., 2001; Vickers, Gershenzon, et al., 2009) is not plausible owing to the relatively low concentrations of isoprene in biological membranes.

Using three model systems—Arabidopsis expressing *Eucalyptus globulus ISPS*, tobacco expressing *Populus alba ISPS*, and wild‐type Arabidopsis fumigated with isoprene—we showed that both endogenous exposure to isoprene through constitutive expression of *ISPS* and exogenous exposure to isoprene can change gene expression in plants (Harvey & Sharkey, 2016; Zuo et al., 2019). Results from a fourth system, RNAi poplar trees lacking detectable ISPS have also been published (Behnke et al., 2007, 2010). Therefore, the hypothesis of a novel role for isoprene as a signaling molecule is emerging. Transcriptomic data from above model systems, and transcriptomic and proteomic data from native isoprene emitters like grey poplar (*Populus* × *canescens*) show that isoprene can alter expression of genes important for growth regulator biosynthesis and signaling, and those important for resilience against abiotic and biotic stress, and growth in plants (Behnke et al., 2010; Dani et al., 2022; Harvey & Sharkey, 2016; Monson et al., 2021; Zuo et al., 2019).

Interestingly, although isoprene‐emitting plants are widespread across the plant kingdom, not all plants make isoprene (Hanson et al., 1999; Monson et al., 2013; Sharkey et al., 2001, 2013). The scattered distribution of isoprene emission among land plants indicates relatively frequent gains and losses of *ISPS*, a TPS‐b terpene synthase unique to angiosperms (Sharkey et al., 2013). Acyclic monoterpene synthases are similar to ISPS in terms of substrate shape and reaction mechanism. Therefore, changes in a few amino acids could convert the former to ISPS (Li et al., 2017; Sharkey et al., 2013). When non‐emitting plants like Arabidopsis and tobacco are genetically engineered to emit isoprene, the regulation of isoprene emission under varying environmental conditions is similar to that in native isoprene emitters (Vickers, Possell, et al., 2009; Zuo et al., 2019). This shows that the regulatory mechanism of isoprene is not specific to isoprene itself but related to inherent regulatory mechanisms of the MEP pathway. Similarly, based on evidence from recent transcriptomic studies (Zuo et al., 2019), we propose that one of the reasons that enhances the likelihood of evolution of the trait of isoprene emission is the fact that isoprene utilizes existing stress signaling networks for signaling purposes. For example, it is likely that isoprene recruits transcription factors (TFs) involved in abiotic stress responses for signal transduction. Some important TFs affected by isoprene include MYBs 9, 59, 73, S2; WRKYs 31 and 40; ERFs 13, 105, and 109; and ATAF1 (Harvey & Sharkey, 2016; Zuo et al., 2019). However, not all TFs are likely to be detected during gene expression studies, and characterization of important cis‐regulatory elements (CREs) in promoters of isoprene‐responsive genes and their corresponding TFs is yet to be done.

**FIGURE 1:**
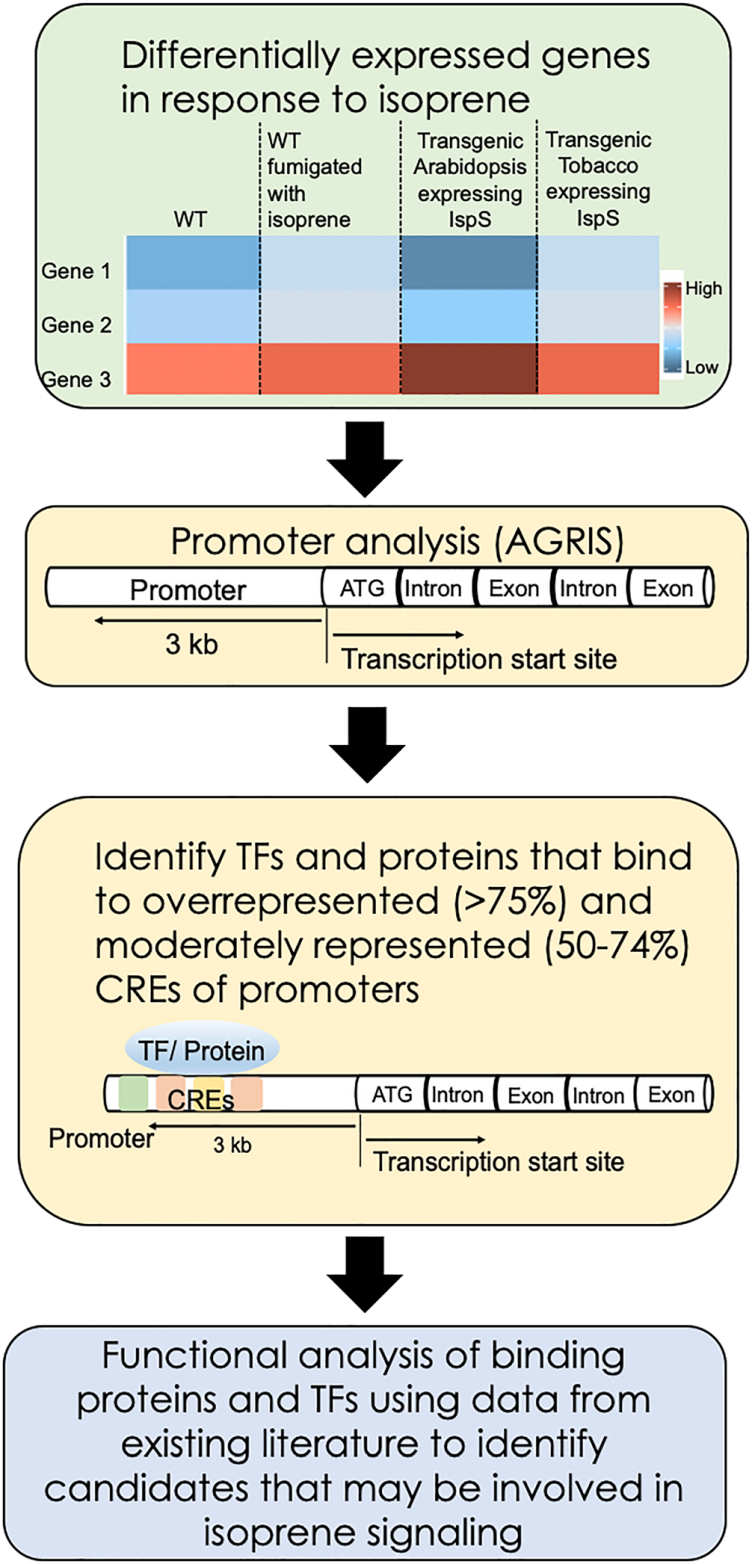
Schematic workflow of bioinformatic analysis of cis‐regulatory elements (CREs) present in promoters of isoprene‐responsive genes to identify TFs and other proteins involved in the isoprene‐signaling pathway. CREs, cis‐regulatory elements.

During the present study, we carried out a comprehensive bioinformatics analysis of important CREs present in promoters of isoprene‐responsive genes to obtain clues about the TFs and other proteins involved in the isoprene‐signaling pathway (Figure 1). We found that promoters of isoprene‐responsive genes were most enriched in CREs that bind TFs such as ARR1, Dof, DPBF, bHLH112, GATA factors, GT‐1, MYBs, and WRKYs; light, abscisic acid (ABA), and JA responsive elements were also overrepresented. More than 50% of isoprene‐responsive genes were found to possess CREs for class IV homeodomain leucine zipper (HD‐ZIP) family TFs that have the ability to bind lipids. Therefore, likelihood of isoprene to interact with HDG11, an important member of this TF‐family, was also explored during the present study.

## 2 MATERIALS AND METHODS

### 2.1 Analysis of CREs present in promoters of isoprene‐responsive genes

We used the list of isoprene‐responsive genes described in Zuo et al. (2019) (Table S1 in the supporting information). This list of genes includes differentially expressed genes in response to isoprene, found to be common to two or all three of the following model systems: *Arabidopsis thaliana* wild‐type (Col‐0) fumigated with 20 μl L^−1^ isoprene for 24 h, and (2) Arabidopsis and tobacco engineered to emit isoprene. The 20 μl L^−1^ isoprene is the estimated concentration of isoprene in mesophyll cells of highly emitting leaves (Singsaas et al., 1997). To identify CREs in the promoter regions of isoprene‐responsive genes, the Arabidopsis CRE database (https://agris-knowledgebase.org/AtcisDB/) (Palaniswamy et al., 2006; Yilmaz et al., 2010) was used to analyze the upstream 3‐kb promoter region of genes (Davuluri et al., 2003). Information on TFs and other proteins that bind to each promoter motif and their functional characteristics was determined using AGRIS and literature surveys. A CRE was considered overrepresented or moderately overrepresented if ≥75% or 50–74% of promoters, respectively, were enriched in that motif. A promoter motif was considered underrepresented if the motif was present in ≤49% of promoters that were analyzed.

### 2.2 *In‐silico* molecular simulation and modeling to test the capacity of HDG11 protein to bind to isoprene as well as to the thylakoid membrane

Fifty percent or more of isoprene‐responsive genes possessed CREs targeted by the class IV HD‐ZIP family steroidogenic acute regulatory protein‐related lipid transfer (START)‐domain‐containing TFs. These TFs have the ability to bind lipids through the START domain. HDG11 is a START domain protein. It transactivates stress responsive genes, which are also responsive to isoprene. Therefore, we selected HDG11 to test *in‐silico* the ability of isoprene to bind to the START domain proteins. Molecular simulation and modeling techniques were combined to develop an atomic model for the START domain within the homeobox‐leucine zipper protein HDG11 (uniprot code Q9FX31) (UniProt, 2015). In turn, this model was tested for its capacity to bind to isoprene as well as to thylakoid membrane models. The initial protein structure was homology modeled with the RoseTTAfold web server (Baek et al., 2021) and solvated in explicit water. The initial model was further refined and sampled using the temperature replica exchange method TIGER2h (Kulke et al., 2018) using NAMD 3.0a9 (Phillips et al., 2020), which facilitates extensive conformational sampling to identify the most probable protein conformations to which isoprene may bind. A detailed description of the methodology is given in the supporting information.

## 3 RESULTS

### 3.1 Characterization of promoter elements of isoprene‐responsive genes

#### 3.1.1 CREs overrepresented in promoters of genes responsive to isoprene

Expression of 61 and 54 genes was found to be upregulated and downregulated, respectively, in response to isoprene in the three plants systems used by Zuo et al. (2019) (Table S1). A total of 226 promoter motifs were represented in these genes which belonged to 145 main CRE groups.

Twenty‐two overrepresented CREs were identified in genes upregulated in response to isoprene (Table 1). Ten CREs were overrepresented in genes downregulated in response to isoprene, out of which seven CREs (DRE‐like motif, GATA box, MYB‐binding site, W box, I Box, RAV1‐binding site, and SORLIP1 motif) were common with those seen in upregulated genes. CREs that bind ARR1, Dof, DPBF, bHLH112, GATA factors, GT‐1, MYB, and WRKY TFs were found in promoters of all genes upregulated in response to isoprene. Also, while the LFY, ATB2, T Box motifs were overrepresented in the genes downregulated in response to isoprene, these were moderately overrepresented in upregulated genes.

**TABLE 1:** CREs overrepresented in promoters of isoprene‐responsive genes. The number of isoprene‐responsive genes carrying a specific CRE is given as a percent of the total number of isoprene‐responsive genes.

CRE	Interacting TF or protein	Sequence of binding motif	Percent of upregulated genes with CRE	Percent of downregulated genes with CRE
ARR1‐binding site	ARR1	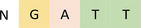	100	
Dof‐binding site	Dof	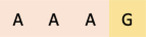	100	
DRE‐like motif	DPBF‐1, DPBF‐2	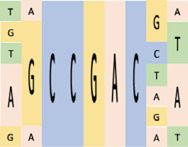	100	96
E box	bHLH112	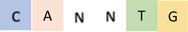	100	
GATA box	GATA factors	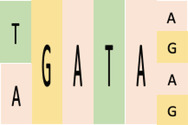	100	94
GT1‐binding site	GT‐1	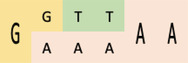	100	
MYB‐binding site	MYB1	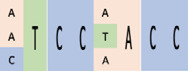	100	92
	MYB2	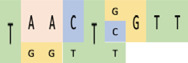		
	MYB3	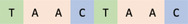		
	MYB4	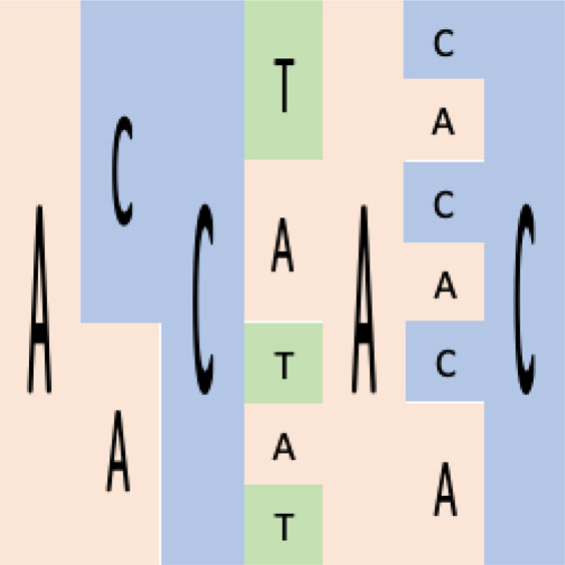		
	MYB26	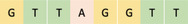		
SEF‐binding motif	SEF1	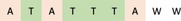	100	
	SEF3	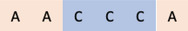		
	SEF4	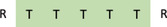		
W BOX	WRKY	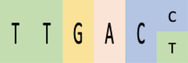	100	75
ROOTMOTIFT‐APOX1	‐	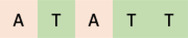	98	
OSE1/2ROOT‐ NODULE	‐	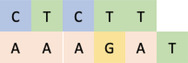	97	
CACTFTPPCA1	‐	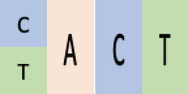	95	
GTGA motif		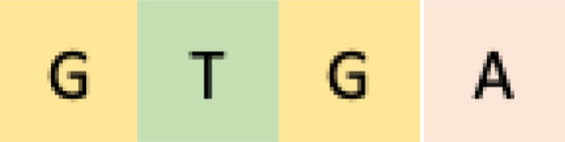	95	
I box	I box binding factors	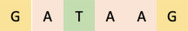	95	75
POLLEN1 LELAT52	‐	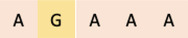	95	
RAV1‐binding site	RAV1	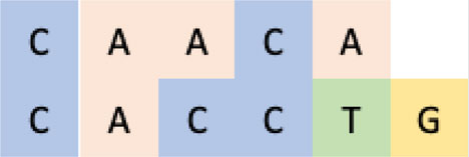	95	90
BIHD1	BIHD1	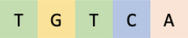	84	
MYC‐recognition site	MYC2, ICE1	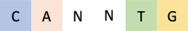	84	73
CCAAT BOX 1	‐	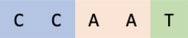	82	
EEC Consensus motif	LCR1	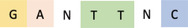	82	
SORLIP	SORLIP1	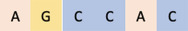	79	84
	SORLIP2	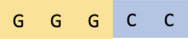		
	SORLIP5	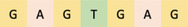		
ACGT box	bZIP factors	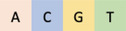	75	
LFY	LFY	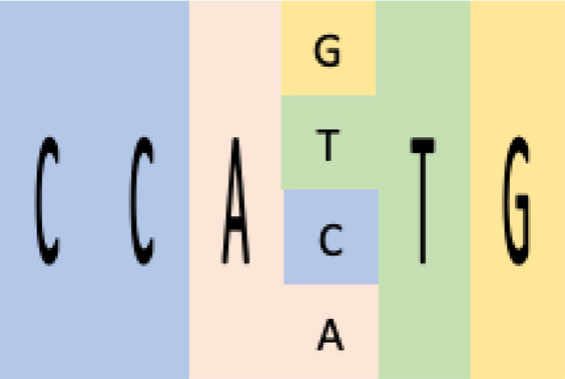	70	75
T Box	bHLH	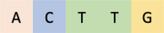	69	75
ATB2 motif	ATB2 bZIP	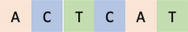	67	75

*Note*: ≥75% of promoters were enriched in that motif for upregulated genes or downregulated genes or both groups. CREs, cis‐regulatory elements; TF, transcription factor.

**FIGURE 2:**
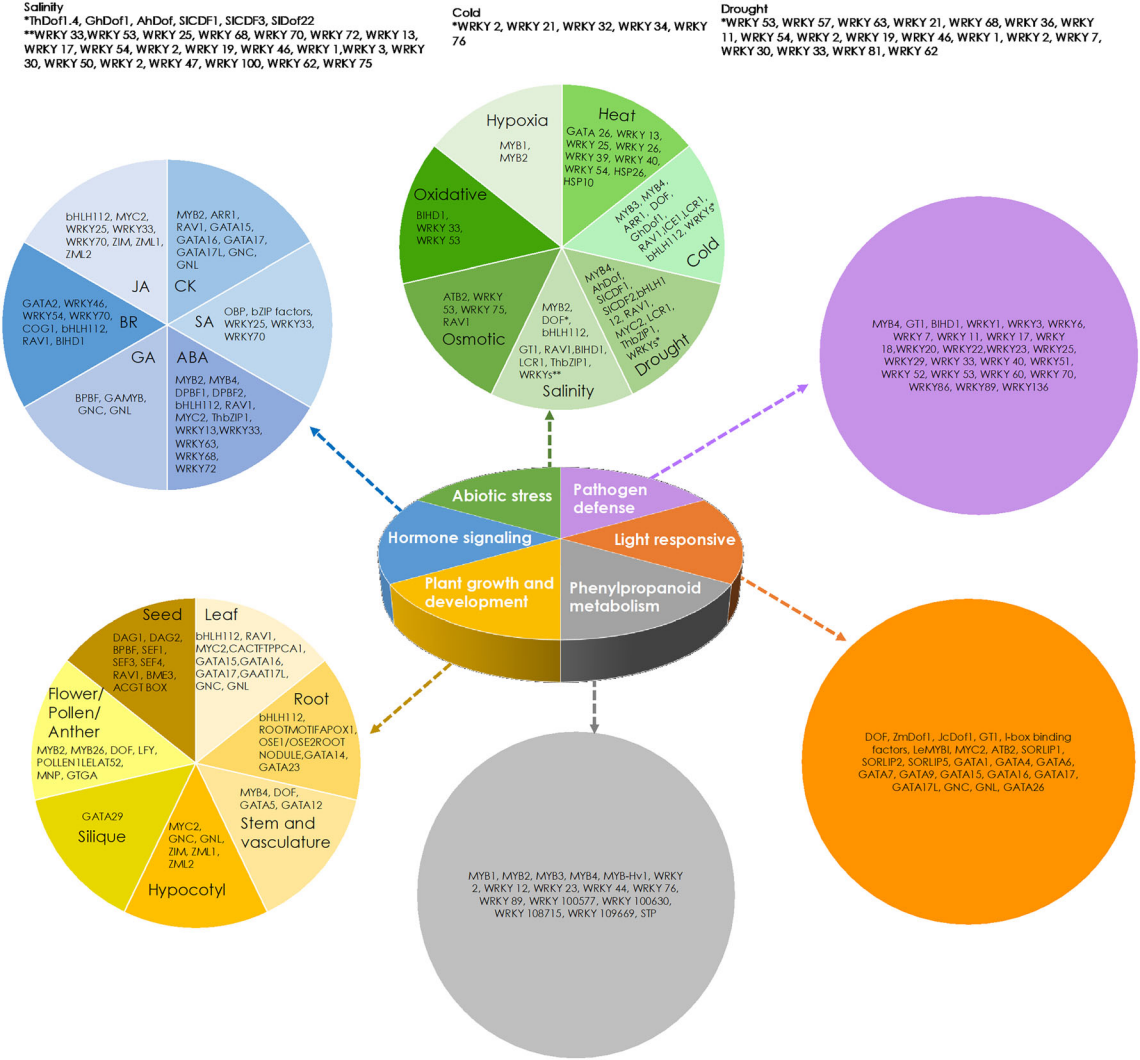
Functions of proteins/transcription factors that are known to bind to CREs of isoprene‐responsive genes and may be related to isoprene signaling. CREs, cis‐regulatory elements.

The CREs present in more than 75% of genes responsive to isoprene, included those responsive to ABA (DRE‐like motif, E‐box, MYB‐binding site, RAV1‐binding site, MYC‐recognition site, ACGT box, and W box), JA (E box, MYC‐recognition site, W box, GATA box), SA (DOF binding site, W box, and ACGT box), CK (ARR1‐binding motif, GATA box, RAV1‐binding site, and MYB binding site), GA (DOF binding site, GATA Box), and brassinosteroid (BIHD1 binding site, RAV1 binding site, E box, DOF bonding site, W box, and GATA box) (Figure 2 and Table S2). Interestingly, no motifs that control gene expression in response to auxin were found among CREs overrepresented in isoprene‐responsive genes. This is consistent with previous findings where isoprene was found to enhance JA, CK, and GA signaling (Zuo et al., 2019).

The CREs overrepresented in promoters of genes responsive to isoprene included those controlling gene expressions related to abiotic stresses such as drought (MYB binding site, DOF binding site, E box, RAV1 binding site, MYC2 recognition site, EEC motif, W box, ACGT box), salt (MYB binding site, DOF binding site, E box, GT1 binding site, RAV1 binding site, BIHD1, EEC motif, W box, and ACGT box), osmotic (RAV1 binding site and W box), oxidative (BIHD1 and W box), heat (GATA box, W box, and CCAAT box), cold (MYB binding site, DOF binding site, ARR1 binding site, RAV1 binding site, E box, W box, MYC2 recognition site, and EEC motif), and hypoxia (MYB binding site) (Figure 2 and Table S2). Four different CREs related to defense against pathogens and herbivory (MYB binding site, GT1 binding site, BIHD1, W box) were overrepresented in promoters of isoprene‐responsive genes. Although MYB binding site and W box were seen in both upregulated and downregulated genes, GT1 and BIHD1 binding sites were seen only in the upregulated genes.

In general, DOF binding site, GT1 binding site, GATA box, MYC recognition site, I box, ATB2, and SORLIP1 motifs, which are light responsive elements, were overrepresented in isoprene‐responsive genes (Table 1). Light responsive elements such as DOF binding site and GT1 binding site were overrepresented only in the upregulated genes, whereas the others were common to both upregulated and downregulated genes. Furthermore, overrepresented promoter motifs like MYB binding site, DOF binding site, CCAT box, and W box are also known to be involved in phenylpropanoid metabolism. In addition, several promoter motifs important for plant growth and development were also overrepresented in isoprene‐responsive genes. These include motifs known to be involved in seed germination and development, leaf development and senescence, chloroplast growth, division and development, stomatal development, root growth, and floral development (Table S2). Many of these motifs show root‐specific (ROOTMOTIFAPOX‐1), anther‐specific (GTGA), and pollen‐specific (GTGA, POLLEN1 LELAT52) expression.

#### 3.1.2 CREs moderately overrepresented in promoters of genes responsive to isoprene

Although 19 moderately overrepresented CREs were identified in promoters of genes upregulated in response to isoprene, only five were found in downregulated genes (Table S3). Similar to overrepresented CREs, motifs related to hormone signaling, biotic and abiotic stress, and light responsive elements were found in the moderately overrepresented CREs. A number of ABA responsive motifs (ABRE‐like‐binding site, Reα, HD‐Zip‐binding elements, TATCCAY motif, and MYC‐recognition site) were seen in the moderately overrepresented CREs in promoters of isoprene‐responsive genes (Table S3). Four CREs that control gene expression in response to auxin were also found (SURE sulfur‐responsive element, CArG motif, BBF1‐binding motif, and ASF‐1 binding site).

#### 3.1.3 CREs underrepresented in promoters of genes responsive to isoprene

In general, CREs underrepresented in promoters of upregulated genes were also underrepresented in downregulated genes (Table S4). Some interesting examples are ATHB‐, HSF‐, CBF‐, and TGA1‐binding site motifs. Although BOX II was moderately overrepresented in downregulated genes, it was underrepresented in upregulated genes. In addition, ABRE‐like‐binding site motif, CArG motif, and LTRE motifs were underrepresented in downregulated genes, whereas they were moderately overrepresented in upregulated genes.

### 3.2 Ability of isoprene to bind to class IV homeodomain HD‐ZIP family START domain containing proteins

In light of the above results, we attempted to find possible receptors for isoprene. More than half of the isoprene‐responsive genes contained at least one binding site (Bellringer/replumless/pennywise BS1 IN AG, Bellringer/replumless/pennywise BS1 IN AG, L1 box) for TFs of the class IV homeodomain HD‐ZIP family, such as GL2, ATML1, PDF2, HDG11, and ATHB17 (Table 2). Although the HD‐zipper‐loop‐zipper (ZLZ) domain binds to the L1 box of the promoter region, a special domain called the steroidogenic acute regulatory protein‐related lipid transfer or START domain can bind ligands such as fatty acids (e.g., linolenic and linoleic acid) (Khosla, 2015; Schrick et al., 2004, 2014). Given that isoprene is an unsaturated hydrocarbon similar to tails of linolenic and linoleic acid, we hypothesized that isoprene binds to the active site of above START proteins. HDG11 transcription factor has been shown to activate the transcription of genes related to JA synthesis and signaling, ABA synthesis, and stress tolerance; some of these genes were also upregulated in response to isoprene. Therefore, we hypothesized that isoprene may be changing gene expression in part by binding to HDG11. To test this, computational simulations were performed to model interactions between isoprene and HDG11 within the thylakoid membrane.

**TABLE 2:** CREs moderately overrepresented in promoters of isoprene‐responsive genes. The number of isoprene‐responsive genes carrying a specific CRE is given as a percent of the total number of isoprene‐responsive genes. Moderately overrepresented: 50–74% of promoters were enriched in that motif. The presence of a green, orange, or yellow box indicates that the CRE and associated TFs control growth (G), stress (S), or light (L)‐related responses, respectively. MO, moderately overrepresented; UR, underrepresented.

CRE	Interacting TF or protein	Functional significance of the corresponding TF binding to CRE	G	S	L	Percent of upregulated genes with CRE	Percent of downregulated genes with CRE
ABRE‐like‐binding site motif	TRAB1, ABFs	Mediates **ABA**‐induced transcription and drought stress tolerance.				74	
REα	Phytochrome	Motif required for **phytochrome** regulation of expression; important in **ABA** and/or light stress signaling pathways.				72	
CuRE	CRR1 SPL gene family	Transcriptional activation of copper‐deficiency target genes.				70	
LFY‐binding site motif	LFY	Controls transition from vegetative to reproductive development.				70	
Pyrimidine box	BPBF	**GA**‐induced activator of reserve protein genes during development; control of hydrolase genes following seed germination.				69	
GARE (GA‐ responsive element)	YAB1	Feedback regulation of **GA** biosynthesis in rice.				67	
SURE	ARFs SLIM1	ARF‐controls expression of **auxin** responsive genes. SLIM1‐controls sulfur‐deficiency transcriptional response.				67	
PREAT	ATB2	Transcriptional activator for hypoosmolarity‐inducible gene expression.				67	
CArG motif	AGL15 MADS TF Family	Negatively regulates **auxin** signaling to promote somatic embryogenesis.				66	
MARTBOX	‐	Control of scaffold attachment or matrix attachment region.				64	
ARF element	ARFs	Regulation of **auxin**‐responsive genes.				64	69
‐10 PEHVPSBD		Motif important for light regulation and circadian cycling.				59	
AMYBOX1 (Sugar starvation enhancer element)		Motif important for negative regulation of genes by sugar.				57	
HD‐ZIP binding cis‐elements (L1 box)	GL2, ATML1, PDF2, HDG11, ATHB17, HD‐ZIP IV TF	Modulates root hair initiation and trichrome branching; Induced by **ABA**, ROS, drought, and NaCl. Positive regulators of salt, drought, oxidative stress				57	65
LTRE promoter motif	AP2/ERF	Putative low‐temperature‐responsive element.				57	
CPB‐BINDING motif	CPB	Controls **CK** responsive gene expression.				56	
BBF1‐binding motif	BBF1	Upregulates genes involved in virus resistance and pathogen defense; regulates **auxin** inducible gene expression.				56	
TATCCAY motif	MYBS1, S2, S3	Regulates glucose and **ABA** signaling during seed germination and early seedling development.				52	
ASF‐1 binding site	ASF‐1, TGA‐1, TGA‐6	Involved in **auxin** and/or **SA** mediated in transcriptional activation.				51	
MYC‐recognition site	MYC2	Regulates **JA**‐responsive gene expression; oxidative stress tolerance; freezing tolerance; osmotic stress tolerance; induced by drought, salinity, A**BA**.				OR	73
Box II promoter motif	CPRF‐1,‐2,‐3	Associated with light stress, controls phloem specific gene expression				UR	71
CCA1 binding site motif	MYB‐related TF	Critical for circadian control and **phytochrome** regulation.				UR	57

*Note*: CREs, cis‐regulatory elements; TF, transcription factor; HD‐ZIP, homeodomain leucine zipper.

**FIGURE 3:**
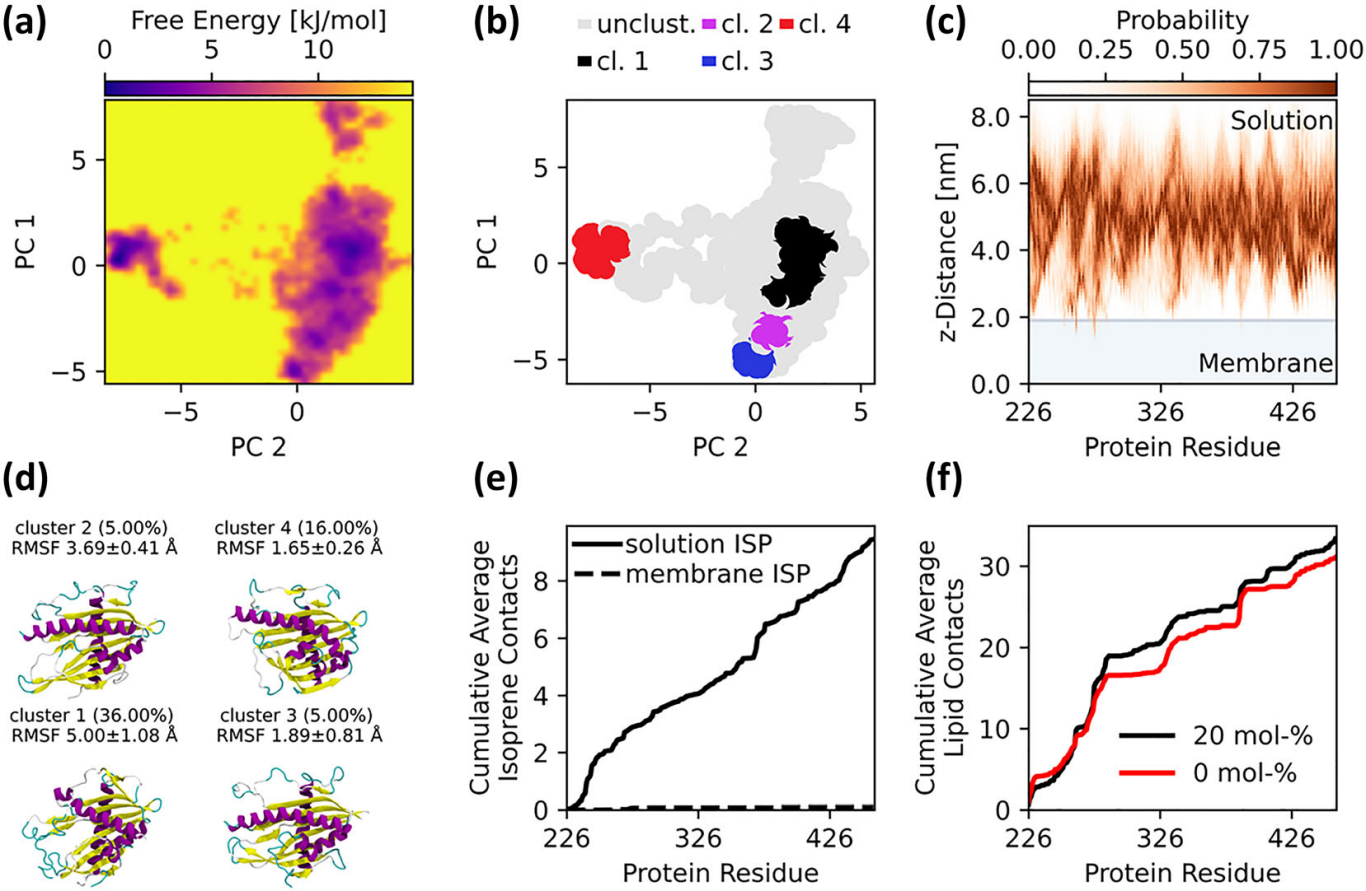
(a) Folding free energy landscape projection from the replica exchange simulation along the first two principal components. Each point depicts a unique protein conformation. The coloration indicates the free energy from *black*, the free energy minimum, to *yellow,* unsampled high energy states. (b) Cluster analysis of the free energy landscape with (d) depicting representative structures, the probability seen from simulation to be within a given cluster, and the root‐mean‐square fluctuation (RMSF) for structures within the cluster. The cluster center conformations within Figure 3d are shown in cartoon representations colored by the secondary structure elements *yellow* β‐sheet, *purple* α‐helix, *cyan* turn, and *white* random coil. (c) HDG11 START domain residue penetration depth into the thylakoid membrane. The blue shaded area shows the membrane area defined as the area within the phosphate atoms of the lipid head groups, whereas the protein residue distances in relation to the membrane center are red‐shaded histograms. Dark red regions demonstrate the highest likelihood to find protein residues at the respective distance from the membrane center. (e) Contacts between HDG11 START domain protein residues and isoprene molecules averaged over the trajectory frames. The graph differentiates between isoprene in *solid line* aqueous solution and *dashed line* inside the membrane. (f) The same as Figure 3e but for the contacts between protein residues and lipids in thylakoid membranes containing 0 mol% (*red*) and 20 mol% (*black*) isoprene.

Quantification of direct HDG11‐isoprene interactions was approached computationally. The START domain was a particularly attractive target, given the role of the START domain in binding to lipophilic substrates. To date, there exists no experimentally determined structure specific to the START domain in HDG11. However, because of their ubiquity (Jentsch et al., 2018; Kudo et al., 2008; Nakao et al., 2019) and structural conservation across species (Schrick et al., 2004, 2014), analogous mammalian START domains are a reasonable starting point to generate homology models. Because of the low sequence identity between potential templates, the resulting model structure was subsequently refined with replica exchange simulations to sample the conformational free energy landscape for the protein (Figure 3a) to identify the minimum free energy structure of the protein. Four clusters were identified within the resulting free energy landscape (Figure 3b), with the free energy minimum located in cluster 4 (Figure 3a). Cluster 4 represents in our view the best description for the HDG11 START domain, as it encompasses the minimum free energy state. Although cluster 4 does not have the highest state probability (Figure 3d), accounting for only 16% of the sampled configurations, the conformations sampled in cluster 4 exhibited less variability between cluster members. By contrast, the most populous cluster, cluster 1, had substantially larger fluctuations around the mean geometry. Thus, we use the model from cluster 4 as our basis for isoprene binding simulations.

By placing the START domain of HDG11 above a membrane in both the presence and absence of isoprene, we are simultaneously asking if the START domain will interact with the thylakoid membrane and, if so, if isoprene can directly modulate the START domain. From the molecular simulation trajectories, protein residues did not typically come into close contact with the membrane (Figure 3c). Instead, the START domain as a whole was typically soluble, with typical residues nearly 3 nm from the membrane surface. Occasionally, the START domain drifted towards the membrane surface and briefly interacted with the thylakoid membrane before diffusion away again, producing measurable but fleeting contacts (Figure 3f). For other peripheral membrane proteins, the number of contacts measured by the same metric can be 10 times larger (Vermaas & Tajkhorshid, 2017). Protein–lipid interaction was not influenced by isoprene concentration.

Membrane binding for isoprene‐interacting proteins is likely essential. During simulation, isoprene molecules accumulate inside the thylakoid membrane, with a substantially lower concentration of isoprene in the aqueous phase (Figure S1). This agrees with earlier findings in DMPC bilayers simulation studies (Siwko et al., 2007), as well as measured partition coefficients for isoprene (Filser et al., 1996; Gargas et al., 1989). For HDG11, or any other potential isoprene‐active protein, membrane association would facilitate isoprene interaction by increasing local isoprene concentration in the protein environment. This was not observed during our simulation. Only short interactions between the START domain and the thylakoid membrane were observed, rather than deep membrane penetration by START domain residues (Figure 3c). Without membrane penetration by the START domain, isoprene within the membrane bilayer was inaccessible to this domain, preventing protein interactions with membrane bound isoprene (Figure 3e). The low isoprene concentration in the aqueous phase engaged in short, nonspecific interactions with the START domain. In summary, a significant interaction between isoprene within the membrane bilayer and the START domain of HDG11 could not be detected, owing to short interactions between the START domain of HDG11 and the thylakoid membrane where isoprene predominantly accumulates.

## 4 DISCUSSION

Transcriptomics and some proteomics studies have indicated that the protective role of isoprene against abiotic and biotic stresses may be through isoprene‐mediated stress signaling leading to changes in gene expression (Behnke et al., 2010; Dani et al., 2022; Harvey & Sharkey, 2016; Monson et al., 2021; Zuo et al., 2019). However, proteins involved in the putative isoprene‐signaling pathway are not known. The goal of the present study was to characterize the CREs present in isoprene‐responsive genes in order to obtain more information on the TFs likely to bind these CREs, and are therefore, involved in isoprene signaling. We also investigated the possibility of isoprene to bind START‐domain‐TFs.

### 4.1 CREs of promoters of isoprene‐responsive genes provide insights into how isoprene controls gene expression

The fact that promoters of genes upregulated in response to isoprene carried a wider variety of CREs than the downregulated genes indicates that upregulated genes have more binding sites that make these genes subjects of more complex regulation than downregulated genes. Collectively, CREs overrepresented in promoters of isoprene‐responsive genes showed that TFs involved in ABA‐, JA‐, and SA‐signaling, and correspondingly involved in drought‐, salt/osmotic‐, oxidative‐, and herbivory/wounding/pathogen‐stress related signaling most likely play a major role in the isoprene‐signaling pathway. Analyses of the CREs of isoprene‐responsive genes showed that these genes are also light responsive. The *ISPS* promoter region contains CREs that are regulated by light and the circadian clock (Parveen et al., 2019). This is consistent with the observation of a very strong circadian variation in mRNA for isoprene synthase, even in constant light (Mayrhofer et al., 2005). This provides evidence that *ISPS* and isoprene‐responsive genes are likely part of the light responsive transcriptome.

It is interesting that CBF‐, HSF‐, WUS‐, and TGA1‐binding motifs were found to be underrepresented in promoters of isoprene‐responsive genes. CBF TFs play an important role in cold tolerance (Park et al., 2015), whereas HSFs are important for heat responses and adaptation (Zhao et al., 2020). As previous reports have shown that isoprene can protect plants from heat stress (Behnke et al., 2007, 2010; Loivamäki et al., 2007; Pollastri et al., 2014; Sasaki et al., 2007; Sharkey et al., 2008; Sun et al., 2013; Velikova & Loreto, 2005), we hypothesized that isoprene‐responsive genes may carry the HSF motif. It is possible that isoprene‐mediated tolerance to heat stress may not be through targeting HSF elements of genes important for heat stress tolerance, but through oxidative stress‐related elements, because promoters of isoprene‐responsive genes were enriched in CREs important to drive expression of genes important for oxidative stress tolerance. WUS is a TF that suppresses stem cell proliferation (Ikeda et al., 2009), and TGA1 is important for SA‐mediated gene expression (Lindermayr et al., 2010).

During previous studies, we showed that isoprene can alter expression of important TFs such as MYB, WRKY, and ERFs (Harvey & Sharkey, 2016; Zuo et al., 2019) which likely act upon other isoprene‐responsive genes further downstream in the signaling cascade of isoprene. Analyses of CREs of promoters of isoprene‐responsive genes and the corresponding TFs that bind to these motifs revealed that most isoprene‐responsive genes also carry binding motifs for these TFs. For example, as mentioned above, expression of enzymes important for phenylpropanoid biosynthesis and regulation (*PAL1*, *PAL2*, *PAL4*, *4CLI1*, *4CLI2*, *4CLI3*, *CCOAMT*) were upregulated in Arabidopsis fumigated with isoprene and in transgenic tobacco engineered to emit isoprene (Harvey & Sharkey, 2016; Zuo et al., 2019). The current promoter analysis revealed that promoters of all these genes have MYB binding sites. Therefore, it is likely that isoprene utilizes MYB, WRKY, and ERFs TFs and corresponding CREs in promoters of isoprene‐responsive genes to carry out its signaling. Furthermore, all the upregulated genes and 94% of the downregulated genes showed the presence of GATA box in their promoters. GATA factors that bind to this motif are involved in a wide variety of functions that include plant growth and development (GATA 2, GATA 5, GATA 12, GATA 15, GATA 16, GATA 17, GATA 17l, GATA 23, ZML1, ZML2, ZIM, BME3, HAN, HNL1, and HNL2) (De Rybel et al., 2010; Endo et al., 2015; Luo et al., 2010; Nawy et al., 2010; Ranftl et al., 2016; Shikata et al., 2004; Zhang et al., 2013; Zhao et al., 2004), photoprotection (ZML1, ZML2, and ZIM) (Shaikhali et al., 2012), chloroplast development (GNC, GNL, GATA 15, GATA 16, GATA 17, GATA 17l, and GATA 26) (An et al., 2020; Behringer & Schwechheimer, 2015; Bi et al., 2005; Chiang et al., 2012; Hudson et al., 2011), and JA signaling (ZML1, ZML2, ZIM) (Chini et al., 2009; Chung & Howe, 2009). Although the role of isoprene in some of these functions like photoprotection has been explored (Pollastri et al., 2019), and its involvement in chloroplast development and JA signaling remains an interesting topic for future research.

Although some of the CREs were common in promoters of both upregulated and downregulated genes, there were a few that were observed only in the upregulated genes. For example, all of the upregulated genes contained the ARR1 binding site. ARR1 is a transcriptional activator of genes responsive to cytokinin. This is consistent with Dani et al. (2021) who showed enhanced CK‐synthesis, CK‐signaling, and CK‐degradation gene expression in isoprene‐emitting Arabidopsis. Similarly, AP2/ERF family of transcription factors that bind to the LTRE promoter element was observed only in the genes upregulated by isoprene. ERF2, a member of this TF family, is a positive regulator of JA‐responsive defense genes which supports previous study that showed enhancement of JA signaling in response to isoprene (Zuo et al., 2019).

We hypothesize that a receptor that is directly induced by isoprene likely generates a signaling cascade that interacts/activates downstream TFs that ultimately bind to the CREs in the promoters of isoprene‐responsive genes. As seen during the current promoter analysis, these TFs are also common components of other important growth regulator and stress signaling pathways. The cross‐talk between isoprene‐mediated signaling and other growth regulator and abiotic and biotic stress signaling pathways, in terms of common TFs as seen in this study, is likely to enhance the stability of the isoprene emission trait when it evolves in a plant. The protein binding simulations to isoprene containing membranes suggest that meaningful isoprene–protein interaction requires integral or peripheral membrane proteins that insert into the membrane surface and reach the isoprene‐rich membrane center. Otherwise, the aqueous isoprene concentration may allow a regulatory function of isoprene to sensitive receptor proteins. The investigated START domain of HDG11 is probably not such a receptor protein, because the isoprene interactions to the protein were non‐specific and did not highlight a specific binding site.

## AUTHOR CONTRIBUTIONS

**S.M.W.** and **T.D.S.** conceived the original research plan for promoter analysis. **T.D.S.** supervised the research. **S.M.W.** and **A.S.** carried out the promoter analysis. **M.K.** and **J.V.V.** carried out the *in‐silico* protein docking studies. **S.M.W., A.S.,** and **M.K.** wrote the manuscript. **T.D.S.** and **J.V. V.** revised and edited the manuscript.

## CONFLICT OF INTEREST STATEMENT

The authors declare there is no conflicts of interest.

## Supporting information


**Data S1** Supporting Information

**Table S1** List of upregulated and downregulated genes in response to isoprene.
**Table S2** Functions of proteins/transcription factors that bind to overrepresented CREs of isoprene responsive genes that may be related to isoprene signaling.
**Table S3** CREs moderately overrepresented in promoters of isoprene responsive genes.
**Table S4** CREs underrepresented in promoters of isoprene responsive genes.
**Figure S1** Probability density of membrane components and isoprene for thylakoid membranes that contain (left) 0‐mol% and (right) 20‐mol% isoprene.

## Data Availability

All analyzed data are included in the article and its supporting information.
