# Supplementary material for: Characterization of promoter elements of isoprene‐responsive genes and the ability of isoprene to bind START domain transcription factors
Source: Plant Direct. 2023 Jan 31;7(2):e483. doi: 10.1002/pld3.483 (PMC9889695; doi:10.1002/pld3.483)
Supplement: Supplementary file 1 — Data S1 Supporting Information [file PLD3-7-e483-s002.pdf]

Characterization of promoter elements of isoprene-responsive gene, and the ability of isoprene to bind START domain transcription factors

Sarathi M. Weraduwage[1,2*], Abira Sahu[1], , Martin Kulke[1,2], Josh V. Vermaas[1,2], Thomas D. Sharkey[1,2,3,4]

[1]MSU-DOE Plant Research Laboratory, [2]Department of Biochemistry & Molecular Biology, [3]Great Lakes Bioenergy Research Center, [4]Plant Resilience Institute, Michigan State University, East Lansing, MI, 48824, USA. *Current address: Departments of Biology and Biochemistry, Bishop's University, Sherbrooke, QC, J1M IZ7, Canada.

Supplementary Methods

In-silico molecular simulation and modeling to test the capacity of HDG11 protein to bind to isoprene as well as to thylakoid membrane

Methods

HDG11 START domain Model

Molecular simulation and modeling techniques were combined to develop an atomic model for the START domain within the homeobox-leucine zipper protein HDG11 (uniprot code Q9FX31) (UniProt, 2015). In turn, this model was tested for its capacity to bind to isoprene as well as to thylakoid membrane models. The initial protein structure was homology modeled with the RoseTTAfold web server (Baek *et al.*, 2021) and solvated in explicit water. The initial model was further refined and sampled using the temperature replica exchange method TIGER2h (Kulke *et al.*, 2018) using NAMD 3.0a9 (Phillips *et al.*, 2020), which facilitates extensive conformational sampling to identify the most probable protein conformations to which isoprene may bind.

For simulation, protein and ions force field parameters were defined by the CHARMM36m (Huang *et al.*, 2017) force field, while water was described with the TIP3P model (Jorgensen *et al.*, 1983). The simulations were conducted using 4 fs timesteps to increase sampling efficiency. The longer timesteps required hydrogen mass repartitioning, placing 3 amu on each hydrogen to slow down the fastest vibrational modes in the system (Hopkins *et al.*, 2015). All covalent bonds involving hydrogen were constrained with the SETTLE algorithm (Miyamoto & Kollman, 1992) to the optimal length. Periodic boundary conditions were applied to the system and intermolecular interactions were considered up to 10 Å with a switching function starting at 9 Å. Interaction pair list tables were generated every 20 steps

between atom pairs within 16 Å distance. Long range electrostatic interactions were calculated through the particle mesh Ewald (PME) (Essmann *et al.*, 1995) method using a 1 Å grid spacing. Temperature and pressure were controlled by Langevin thermo- and barostats (Grest & Kremer, 1986; Feller *et al.*, 1995) to the respective replica temperature and pressure described by the Antoine-equation (A=8.14019, B=1810.94°C, C=244.485°C, result in Torr (Onken *et al.*, 1989) multiplied by three. The higher pressures prevent water from vaporizing at higher replica temperatures, while liquid water densities do not significantly change with pressure. All atoms were coupled to the temperature bath with a coupling constant of 10 ps^{-1}. The cell volume oscillation period was set to 200 fs assuming friction that reduced this oscillation with a 100 fs decay damping time. The replica exchange TIGER2h simulations (Kulke *et al.*, 2018) utilized 24 temperature replicas spanning 310-450 K. The temperatures for the replicas were assigned logarithmically with base 10 equidistant over the temperature range (310 K, 315.06 K, 320.21 K, 325.44 K, 330.76 K, 336.16 K, 341.65 K, 347.23 K, 352.9 K, 358.67 K, 364.53 K, 370.48 K, 376.54 K, 382.69 K, 388.94 K, 395.29 K, 401.75 K, 408.31 K, 414.98 K, 421.76 K, 428.65 K, 435.65 K, 442.77 K and 450 K). The TIGER2h method employs heating and cooling simulation cycles. After sampling the protein in explicit solvent for 16 ps, all replicas were cooled to 310 K within 4 ps. Exchanges were evaluated based on the Metropolis sampling criterion (Metropolis *et al.*, 1953) for the protein conformations at 310 K in implicit solvent calling OpenMM 8.4.5 (Eastman *et al.*, 2017). Afterwards, temperatures were reassigned and the next cycle continues.

The protein free energy landscape is constructed from the TIGER2h simulation trajectories by investigating the structure similarity between conformations with dihedral principal component analysis (dPCA) (Altis *et al.*, 2007). dPCA, in contrast to typical principal component analysis in cartesian coordinate space, utilizes the rotationally and translationally invariant backbone dihedral angles ϕ and ψ to describe internal protein conformation. The first two principal components collective encode 25% of the variation seen across protein conformations within the TIGER2h simulations. The resulting two-dimensional density is clustered with the density-based clustering algorithm DBSCAN (Ester *et al.*, 1996) included in the scikit-learn library (Pedregosa *et al.*, 2011). During the clustering, two sample points were considered neighboring up to a distance of 0.5 in the parameter space formed by the first two principal components and density clusters contained at least 600 sample points.

HDG11 binding studies to thylakoid membranes

The most likely protein conformations were tested for their ability to interact with biological membranes, where isoprene is expected to partition within a cell. The membrane builder interface on the CHARMM-GUI website (Jo *et al.*, 2008; Wu *et al.*, 2014) was used to generate multiple *Arabidopsis thaliana* thylakoid membrane models. Lipid head group and tail compositions were adjusted according to current

lipidomics results, rounding percentages to whole lipid molecules **(Table S1)** (Block *et al.*, 1983; Block *et al.*, 2007; Fritz *et al.*, 2007) such that each leaflet had 200 lipids. 60 membranes were prepared in total, varying only the initially randomized lipid starting positions. The concentration of isoprene in thylakoid membranes has been reported to be 0.0044 mol% under physiological conditions. In 30 of these membranes, an excess of 20-mol% isoprene was introduced by substituting water molecules for isoprene. All membrane systems were equilibrated for 200 ns before proceeding with the protein binding simulations.

The solvated replica exchange structure of HDG11's START domain was oriented above the equilibrated thylakoid membrane in 6 different rotations. The rotations resemble the six faces of an imaginary cube around the protein with either the x, y, z, -x, -y, or -z plane facing towards the membrane surface. To improve the sampling statistics, the positional bias in the initial system state was removed by replicating each protein rotation five times and assigning it one of the prepared membranes with unique lipid starting positions resulting in 30 positionally different simulations. All systems were simulated for 200 ns. Intermolecular interaction distances were changed to consider interactions up to 12 Å with a switching function at 10 Å. The interaction pair list generation frequency was increased to every 100 steps. Temperature and pressure were maintained using Langevin thermo-(Grest & Kremer, 1986) and barostats (Feller *et al.*, 1995) to 298 K and 1 bar, respectively. The thermostat coupling constant was reduced to 1 ps^{-1}. The pressure was adjusted separately in xy and z dimension in a semiisotropic ensemble to account for the different compressibilities of lipids and water molecules. All other simulation parameters are kept identical to the replica exchange simulation.

Contacts and distances were analyzed in VMD 1.9.4 (Humphrey *et al.*, 1996) with scripts calculating the interactions between protein residues and membrane components. An interaction between proteins and membrane components was defined as a sigmoid function of the distance d in Å between heavy atoms.

$$C(d) = {}^{1}\!/_{1 + e^{5(d - 4\text{Å})}}$$

The function counts small distance interactions as 1 with a turning point at 4 Å, at which point interactions still count as fractional contacts to account for the fluid nature of some interactions. For computational efficiency, heavy atom pairs further than 6 Å apart were not included in the overall contact sum. These interactions are ensemble averaged over the trajectories for each amino acid.

Table S1. Membrane lipid compositions and general system parameters.

	Headgroup	Tails	0 mol-% Isoprene	20 mol-% Isoprene
Lipids	MGDG	18:3-16:3	120	120
		18:3-18:3	104	104
		16:0-18:3	4	4
	DGDG	18:3-16:3	8	8
		18:3-18:3	80	80
		16:0-18:3	20	20
	SQDG	16:0-18:3	24	24
		16:0-16:0	4	4
	PG	16:0-18:3	4	4
		18:3-16:1	24	24
	PI	16:0-18:3	4	4
Isoprene			0	100
Water			36385*	36178*
Ions	Sodium		224*	223*
	Chloride		155*	154*
System dimensions [*Å³*]			113.9x113.9x130.3*	113.8x113.8x131.3*

* Values differ slightly between rotations and replicas, depending on the exact geometry

References

Altis A, Nguyen PH, Hegger R, Stock G. 2007. Dihedral angle principal component analysis of molecular dynamics simulations. *J Chem Phys* **126**(24): 244111.

Baek M, DiMaio F, Anishchenko I, Dauparas J, Ovchinnikov S, Lee GR, Wang J, Cong Q, Kinch LN, Schaeffer RD, et al. 2021. Accurate prediction of protein structures and interactions using a three-track neural network. *Science* **373**(6557): 871-876.

Block MA, Dorne AJ, Joyard J, Douce R. 1983. Preparation and characterization of membrane fractions enriched in outer and inner envelope membranes from spinach chloroplasts. II. Biochemical characterization. *Journal of Biological Chemistry* **258**(21): 13281-13286.

Block MA, Douce R, Joyard J, Rolland N. 2007. Chloroplast envelope membranes: a dynamic interface between plastids and the cytosol. *Photosynth Res* **92**(2): 225-244.

Eastman P, Swails J, Chodera JD, McGibbon RT, Zhao Y, Beauchamp KA, Wang LP, Simmonett AC, Harrigan MP, Stern CD, et al. 2017. OpenMM 7: Rapid development of high performance algorithms for molecular dynamics. *PLoS Comput Biol* **13**(7): e1005659.

Essmann U, Perera L, Berkowitz ML, Darden T, Lee H, Pedersen LG. 1995. A smooth particle mesh Ewald method. *The Journal of Chemical Physics* **103**(19): 8577-8593.

Ester M, Kriegel H-P, Sander J, Xu X 1996. A density-based algorithm for discovering clusters in large spatial databases with noise. *Second International Conference on Knowledge Discovery and Data Mining*. Portland, Oregon. 226-231.

Feller SE, Zhang Y, Pastor RW, Brooks BR. 1995. Constant pressure molecular dynamics simulation: The Langevin piston method. *The Journal of Chemical Physics* **103**(11): 4613-4621.

Filser JG, Csanády GA, Denk B, Hartmann M, Kaufmann A, Kessler W, Kreuzer PE, Pütz C, Shen JH, Stei P. 1996. Toxicokinetics of isoprene in rodents and humans. *Toxicology* **113**(1-3): 278-287.

Fritz M, Lokstein H, Hackenberg D, Welti R, Roth M, Zahringer U, Fulda M, Hellmeyer W, Ott C, Wolter FP, et al. 2007. Channeling of eukaryotic diacylglycerol into the biosynthesis of plastidial phosphatidylglycerol. *J Biol Chem* **282**(7): 4613-4625.

Gargas ML, Burgess RJ, Voisard DE, Cason GH, Andersen ME. 1989. Partition coefficients of low-molecular-weight volatile chemicals in various liquids and tissues. *Toxicology and Applied Pharmacology* **98**(1): 87-99.

Grest GS, Kremer K. 1986. Molecular dynamics simulation for polymers in the presence of a heat bath. *Phys Rev A Gen Phys* **33**(5): 3628-3631.

Hopkins CW, Le Grand S, Walker RC, Roitberg AE. 2015. Long-Time-Step Molecular Dynamics through Hydrogen Mass Repartitioning. *J Chem Theory Comput* **11**(4): 1864-1874.

Huang J, Rauscher S, Nawrocki G, Ran T, Feig M, de Groot BL, Grubmuller H, MacKerell AD, Jr. 2017. CHARMM36m: an improved force field for folded and intrinsically disordered proteins. *Nat Methods* **14**(1): 71-73.

Humphrey W, Dalke A, Schulten K. 1996. VMD: Visual molecular dynamics. *Journal of Molecular Graphics* **14**(1): 33-38.

Jentsch J-A, Kiburu I, Pandey K, Timme M, Ramlall T, Levkau B, Wu J, Eliezer D, Boudker O, Menon AK. 2018. Structural basis of sterol binding and transport by a yeast StARkin domain. *Journal of Biological Chemistry* **293**(15): 5522-5531.

Jo S, Kim T, Iyer VG, Im W. 2008. CHARMM-GUI: a web-based graphical user interface for CHARMM. *J Comput Chem* **29**(11): 1859-1865.

Jorgensen WL, Chandrasekhar J, Madura JD, Impey RW, Klein ML. 1983. Comparison of simple potential functions for simulating liquid water. *The Journal of Chemical Physics* **79**(2): 926-935.

Kudo N, Kumagai K, Tomishige N, Yamaji T, Wakatsuki S, Nishijima M, Hanada K, Kato R. 2008. Structural basis for specific lipid recognition by CERT responsible for nonvesicular trafficking of ceramide. *Proceedings of the National Academy of Sciences* **105**(2): 488-493.

Kulke M, Geist N, Moller D, Langel W. 2018. Replica-Based Protein Structure Sampling Methods: Compromising between Explicit and Implicit Solvents. *J Phys Chem B* **122**(29): 7295-7307.

Metropolis N, Rosenbluth AW, Rosenbluth MN, Teller AH, Teller E. 1953. Equation of State Calculations by Fast Computing Machines. *The Journal of Chemical Physics* **21**(6): 1087-1092.

Miyamoto S, Kollman PA. 1992. Settle: An analytical version of the SHAKE and RATTLE algorithm for rigid water models. *Journal of Computational Chemistry* **13**(8): 952-962.

Nakao N, Ueno M, Sakai S, Egawa D, Hanzawa H, Shohei Kawasaki, Kumagai K, Suzuki M, Kobayashi S, Hanada K. 2019. Natural ligand-nonmimetic inhibitors of the lipid-transfer protein CERT. *Commun Chem* **2**: 20-31.

Onken U, Rarey-Nies J, Gmehling J. 1989. The Dortmund Data Bank: A computerized system for retrieval, correlation, and prediction of thermodynamic properties of mixtures. *International Journal of Thermophysics* **10**(3): 739-747.

Pedregosa F, Varoquaux G, Gramfort A, Michel V, Thirion B, Grisel O, Blondel M, Prettenhofer P, Weiss R, Dubourg V, et al. 2011. Scikit-learn: Machine Learning in Python. *Journal of Machine Learning Research* **12**(85): 2825-2830.

Phillips JC, Hardy DJ, Maia JDC, Stone JE, Ribeiro JV, Bernardi RC, Buch R, Fiorin G, Henin J, Jiang W, et al. 2020. Scalable molecular dynamics on CPU and GPU architectures with NAMD. *J Chem Phys* **153**(4): 044130.

Schrick K, Bruno M, Khosla A, Cox PN, Marlatt SA, Roque RA, Nguyen HC, He C, Snyder MP, Singh D, et al. 2014. Shared functions of plant and mammalian StAR-related lipid transfer (START) domains in modulating transcription factor activity. *BMC Biology* **12**(1): 70.

Schrick K, Nguyen D, Karlowski WM, Mayer KFX. 2004. START lipid/sterol-binding domains are amplified in plants and are predominantly associated with homeodomain transcription factors. *Genome Biology* **5**(6): R41.

Siwko ME, Marrink SJ, de Vries AH, Kozubek A, Schoot Uiterkamp AJ, Mark AE. 2007. Does isoprene protect plant membranes from thermal shock? A molecular dynamics study. *Biochim Biophys Acta* **1768**(2): 198-206.

UniProt C. 2015. UniProt: a hub for protein information. *Nucleic Acids Res* **43**(Database issue): D204-212.

Vermaas JV, Tajkhorshid E. 2017. Differential Membrane Binding Mechanics of Synaptotagmin Isoforms Observed in Atomic Detail. *Biochemistry* **56**(1): 281-293.

Wu EL, Cheng X, Jo S, Rui H, Song KC, Davila-Contreras EM, Qi Y, Lee J, Monje-Galvan V, Venable RM, et al. 2014. CHARMM-GUI Membrane Builder toward realistic biological membrane simulations. *J Comput Chem* **35**(27): 1997-2004.
