# Supplementary material for: Characterization of promoter elements of isoprene‐responsive genes and the ability of isoprene to bind START domain transcription factors
Source: Plant Direct. 2023 Jan 31;7(2):e483. doi: 10.1002/pld3.483 (PMC9889695; doi:10.1002/pld3.483)
Supplement: Supplementary file 2 — Table S1 List of upregulated and downregulated genes in response to isoprene. Table S2 Functions of proteins/transcription factors that bind to overrepresented CREs of isoprene responsive genes that may be related to isoprene signaling. Table S3 CREs moderately overrepresented in promoters of isoprene responsive genes. Table S4 CREs underrepresented in promoters of isoprene responsive genes. Figure S1 Probability density of membrane components and isoprene for thylakoid membranes that contain (left) 0‐mol% and (right) 20‐mol% isoprene. [file PLD3-7-e483-s001.docx]

**Characterization of promoter elements of isoprene-responsive genes, and the ability of isoprene to bind START domain transcription factors**

Sarathi M. Weraduwage^1,2,a^, Abira Sahu^1,a^, Martin Kulke^1,2^, Josh V. Vermaas^1,2^, Thomas D. Sharkey^1,2,3,4^

^1^MSU-DOE Plant Research Laboratory, ^2^Department of Biochemistry & Molecular Biology, ^3^Great Lakes Bioenergy Research Center, ^4^Plant Resilience Institute, Michigan State University, East Lansing, MI, 48824, USA.

^a^Co-first authors

**Table S1:** List of upregulated and downregulated genes in response to isoprene

**Table S2:** Functions of proteins/transcription factors that bind to overrepresented CREs of isoprene responsive genes that may be related to isoprene signaling.

**Table S3:** CREs moderately overrepresented in promoters of isoprene responsive genes.

**Table S4:** CREs underrepresented in promoters of isoprene responsive genes.

**Fig. S1:** Probability density of membrane components and isoprene for thylakoid membranes that contain (left) 0-mol% and (right) 20-mol% isoprene.

**Table S1:** List of upregulated and downregulated genes in response to isoprene

| **Upregulated genes** | | **Downregulated genes** | |
| --- | --- | --- | --- |
| **Gene ID** | **Gene** | **Gene ID** | **Gene** |
| AT3G59350 | PTI-1 | AT2G41940 | ZFP8 |
| AT1G64390 | GH9C2 | AT1G19715 | JAL3 |
| AT4G18740 | Rho termination factor | AT1G52000 | JAL5 |
| AT5G55530 | Calcium-dependent lipid-binding (CaLB domain) family protein | AT5G49740 | FRO7 |
| AT1G55020 | LOX1 | AT4G19170 | CCD4 |
| AT1G17420 | LOX3 | AT4G38380 | DTX45 |
| AT1G72520 | LOX4 | AT1G64500 | THRUMIN1 |
| AT3G22400 | LOX5 | AT2G41040 | Methyltransferase |
| AT1G01720 | ATAF1 | AT2G02080 | IDD4 |
| AT1G53430 | NILR | AT4G12290 | CUAO |
| AT1G68710 | ALA9 | AT5G65760 | Serine carboxypeptidase S28 family protein |
| AT1G05010 | ACO4 | AT5G08520 | SRM1 |
| AT3G06130 | ATHMP25 | AT4G24470 | TIFY1 |
| AT1G12110 | NRT1 | AT4G39400 | BRI1 |
| AT1G78080 | WIND1 | AT5G16730 | Microtubule-associated protein |
| AT5G04870 | CPK1 | AT2G25490 | EBF1 |
| AT5G54600 | RPL24 | AT1G30130 | DUF1365 family protein |
| AT5G59680 | Leucine-rich repeat protein kinase family protein | AT5G58770 | ATCPT4, ATCPT7 |
| AT4G24970 | MORC7 | AT1G13940 | T-box transcription factor, putative (DUF863) |
| AT2G19940 | N-acetyl-gamma-glutamyl-phosphate reductase | AT1G30130 | DUF1365 family protein |
| AT1G20780 | PUB44 | AT4G22140 | EBS |
| AT3G47550 | RING/FYVE/PHD zinc finger | AT1G25560 | TEM1 |
| AT5G45030 | Trypsin family protein | AT5G45820 | CIPK20 |
| AT4G18950 | BHP | AT1G63090 | PP2A11 |
| AT5G58260 | NDHN | AT1G72150 | PATL1 |
| AT2G06050 | OPR3 | AT3G19850 | Phototropic-responsive NPH3 family protein |
| AT1G08200 | AXS2 | AT4G37260 | MYB73 |
| AT4G18220 | Drug/metabolite transporter | AT4G38380 | DTX45 |
| AT3G07010 | Pectate lyase-like superfamily protein | AT4G22540 | ORP2A |
| AT1G16670 | CRPK1 | AT5G59320 | LTP3 |
| AT5G48970 | Mitochondrial substrate carrier family protein | AT1G01240 | Transmembrane protein |
| AT5G07020 | MPH1 | AT4G03400 | DFL2 |
| AT1G75350 | EMB2184 | AT5G59780 | MYB59 |
| AT1G03250 | R3H domain protein | AT4G33310 | - |
| AT2G42220 | Rhodanese-like domain protein | AT3G26740 | CCL |
| AT1G77740 | PIP5K2 | AT5G43780 | APS4 |
| AT3G09830 | PCRK1 | AT4G32340 | Tetratricopeptide repeat (TPR)-like superfamily protein |
| AT1G16110 | WAKL6 | AT2G18170 | MPK7 |
| AT5G62570 | CBP60A | AT5G25350 | EBF2 |
| AT4G37370 | CYP81D8 | AT1G19660 | BBD2 |
| AT3G15190.1 | PRPS20 | AT5G44260 | TZF5 |
| AT2G47800 | MRP4 | AT3G05130 | Paramyosin-like protein |
| AT3G21070 | NADK1 | AT5G33290 | XGD1 |
| AT2G16500 | ADC1 | AT3G63210 | MARD1 |
| AT4G34710 | ADC2 | AT5G02760 | APD7 |
| AT5G18550 | ATC3H58 zinc finger -like protein | AT4G24450 | GWD2 |
| AT1G70760 | NDHL | AT1G75380 | BBD1 |
| AT4G01310 | PRPL5 | AT2G03310 | Transmembrane protein |
| AT3G14440 | NCED3 | AT2G21240 | BPC4 |
| AT2G32950 | COP1 | AT3G61060 | ATPP2-A13 |
| AT2G37040 | PAL1 | AT5G63620 | HER2 |
| AT1G51680 | 4CL1 | AT1G80440 | KFB20 |
| AT4G34050 | CCOAMT | AT4G01610 | CATHB3 |
| AT3G06350 | DHQ-SDH |  |  |
| AT4G40090 | AGP3 |  |  |
| AT1G30100 | NCED5 |  |  |
| AT4G02530 | MPH2 |  |  |
| AT3G53260 | PAL2 |  |  |
| AT3G10340 | PAL3 |  |  |
| AT3G21240 | 4CLI2 |  |  |
| AT1G65060 | 4CLI3 |  |  |

**Table S2:** Functions of proteins/transcription factors that bind to overrepresented CREs of isoprene responsive genes that may be related to isoprene signaling.

| **Functions** | **CRE** | **Binding protein/TF** | **Reference** |
| --- | --- | --- | --- |
| **Phenylpropanoid metabolism** | MYB binding site | MYB1 | (1-5) |
|  |  | MYB2 | (6, 7) |
|  |  | MYB3 | (8, 9) |
|  |  | MYB4 | (10-12) |
|  | CCAT box | MYB-Hv1 | (13) |
|  | W box | WRKY2, WRKY12, WRKY23, WRKY44, WRKY76, WRKY89, WRKY100577, WRKY100630, WRKY108715, WRKY109669, STP | (14) |
|  | DOF binding site | AtDOF4;2 | (15) |
| **Light responsive** | DOF binding site | DAG1, DAG2 | (16, 17) |
|  |  | COG1 | (18) |
|  |  | OBP3 | (19, 20) |
|  |  | DOF1 | (21) |
|  |  | JcDOF | (22) |
|  | GT1 binding site | GT1 | (23-26) |
|  | I box binding site | I box binding factor | (27-29) |
|  |  | LeMYBI | (30) |
|  | MYC recognition site | MYC2 | (31) |
|  | ATB2 | bZIP TF | (32) |
|  | SORLIP | SORLIP1 | (33, 34) |
|  |  | SORLIP2 | (34-36) |
|  |  | SORLIP5 | (34) |
|  | GATA box | GATA1, GATA4 | (37) |
|  |  | GATA 6, GATA7, GATA9 | (38) |
|  |  | GATA15, GATA16, GAAT17, GAAT17L | (39) |
|  |  | GNC, GNL | (40) |
|  |  | GATA26 | (41) |
| **Pathogen defense (bacteria, virus, nematode etc.)** | MYB binding site | MYB4 | (42, 43) |
|  | GT1 binding site | GT1 | (25, 44, 45) |
|  | BIHD1 binding site | BIHD1 | (46-48) |
|  | W box | WRKY1, WRKY3, WRKY6, WRKY 7, WRKY 11, WRKY 17, WRKY 18, WRKY20, WRKY22, WRKY23, WRKY25, WRKY29, WRKY 33, WRKY 40, WRKY51, WRKY 52, WRKY 53, WRKY 60, WRKY 70, WRKY86, WRKY89, WRKY136 | (49-53) |
| **Abiotic stress** | | | |
| Heat | CCAT box | HSP10, HSP26 | (54-56) |
| Cold | MYB binding site | MYB3 | (57) |
|  |  | MYB4 | (58, 59) |
|  | ARR1 binding site | ARR1 | (60, 61) |
|  | DOF binding site | DOF | (62) |
|  |  | GhDOF1 | (63) |
|  | RAV1 binding site | RAV1 | (64) |
|  | MYC recognition site | ICE1 | (65-68) |
|  | EEC consensus motif | LCR1 | (69) |
|  | E box | bHLH112 | (70) |
|  | W box | WRKY2, WRKY21, WRKY32, WRKY34, WRKY76 | (71) |
| Drought | MYB binding site | MYB4 | (58) |
|  | DOF binding site | AhDOF1 | (72) |
|  |  | SICDF1, SICDF2 | (73) |
|  | E box | bHLH112 | (74, 75) |
|  | RAV1 binding site | RAV1 | (76) |
|  | MYC recognition site | MYC2 | (77, 78) |
|  |  | ICE1 | (68) |
|  | EEC consensus motif | LCR1 | (79) |
|  | ACGT box | ThbZIP1 | (80, 81) |
|  | W box | WRKY1, WRKY2, WRKY7, WRKY11, WRKY19, WRKY21, WRKY30, WRKY33, WRKY36, WRKY46, WRKY53, WRKY54, WRKY57, WRKY62, WRKY63, WRKY68, WRKY81 | (71) |
| Salinity | MYB binding site | MYB2 | (82, 83) |
|  | DOF binding site | ThDof1.4 | (84) |
|  |  | GhDof1 | (63) |
|  |  | AhDof | (72) |
|  |  | SICDF1, SICDF3 | (73) |
|  |  | SIDof22 | (85) |
|  | E box | bHLH112 | (75) |
|  | GT1 binding site | GT1 | (45) |
|  | RAV1 binding site | RAV1 | (86-88) |
|  | BIHD1 binding site | BIHD1 | (48) |
|  | EEC consensus motif | LCR1 | (79) |
|  | ACGT box | ThbZIP1 | (80, 89) |
|  | W box | WRKY33, WRKY53, WRKY25, WRKY68, WRKY70, WRKY72, WRKY13, WRKY17, WRKY54, WRKY2, WRKY19, WRKY46, WRKY1, WRKY3, WRKY30, WRKY50, WRKY2, WRKY47, WRKY100, WRKY62, WRKY75 | (71) |
| Osmotic | ATB2 | bZIP TFs | (90) |
|  | W box | WRKY53, WRKY75 | (71) |
|  | RAV1 binding site | RAV1 | (86) |
| Oxidative | BIHD1 binding site | BIHD1 | (48) |
|  | W box | WRKY33, WRKY53 | (71) |
| Hypoxia | MYB binding site | MYB1 | (91) |
|  |  | MYB2 | (92) |
| Metal toxicity | MYB binding site | MYB2 | (93) |
|  |  | MYB4 | (94, 95) |
| **Hormone signaling** | | | |
| Jasmonic acid | E box | bHLH112 | (96) |
|  | MYC recognition site | MYC2 | (97-99) |
|  | W box | WRKY25, WRKY33,  WRKY70 | (49) |
|  | GATA box | ZIM, ZML1, ZML2 | (100, 101) |
| Cytokinin | MYB binding site | MYB2 | (102) |
|  | ARR1 binding site | ARR1 | (103-106) |
|  | RAV1 binding site | RAV1 | (107) |
|  | GATA box | GATA15, GATA16, GAAT17, GAAT17L | (39) |
|  |  | GNC, GNL | (40) |
| Salicylic acid | DOF binding site | OBP | (19, 20) |
|  | ACGT box | bZIP factors | (81) |
|  | W box | WRKY25, WRKY33, WRKY70 | (49) |
|  |  |  | (108, 109) |
| Abscisic acid | MYB binding site | MYB2 | (77, 83) |
|  |  | MYB4 | (110) |
|  | DRE like motif | DPBF1, DPBF2 | (111-114) |
|  | E box | bHLH112 | (75) |
|  | RAV1 binding site | RAV1 | (76, 115, 116) |
|  | MYC recognition site | MYC2 | (77, 78, 98) |
|  | ACGT box | ThbZIP1 | (80, 81) |
|  | W box | WRKY13, WRKY33, WRKY63, WRKY68, WRKY72 | (71) |
| Gibberellic acid | DOF binding site | BPBF | (117) |
|  |  | GAMYB | (15) |
|  | GATA box | GNC, GNL | (118) |
| Brassinosteroids | GATA box | GATA2 | (119) |
|  | W box | WRKY46, WRKY54, WRKY70 | (120, 121) |
|  | DOF binding site | COG1 | (122) |
|  | E box | bHLH112 | (123) |
|  | RAV1 binding site | RAV1 | (124) |
|  | BIHD1 binding site | BIHD1 | (46) |
| **Plant growth and development** | | | |
| Seed germination and development | DOF binding site | DAG1, DAG2 | (16) |
|  |  | BPBF | (125) |
|  | SEF binding motif | SEF1, SEF3, SEF4 | (126, 127) |
|  | RAV1 binding site | RAV1 | (128) |
|  | GATA box | BME3 | (129) |
|  | ACGT box |  | (130) |
| Leaf development and senescence | E box | bHLH112 | (123) |
|  | RAV1 binding site | RAV1 | (131) |
|  | CACTFTPPCA1 |  | (132) |
|  | GATA box | GATA15, GATA16, GAAT17, GAAT17L | (39) |
| Stomatal development | MYC recognition site | MYC2 | (133) |
| Chloroplast growth, division, and development | GATA box | GNC, GNL | (40, 134, 135) |
| Root growth and root specific expression | E box | bHLH112 | (136) |
|  | ROOTMOTIFTAPOX1 |  | (137, 138) |
|  | OSE1/OSE2ROOTNODULE |  | (139) |
|  | GATA box | GATA14, GATA23 | (38, 140) |
| Stem and vasculature | MYB recognition site | MYB2 | (141) |
|  | DOF binding site | Dof2.4, Dof5.8 | (142) |
|  | GATA box | GATA5, GATA12 | (143) |
| Hypocotyl | MYC recognition site | MYC2 | (144) |
|  | GATA box | GNC, GNL, ZIM, ZML1, ZML2 | (145, 146) |
| Silique | GATA box | GATA29 | (38) |
| Floral development | DOF binding site | PheDOF2, PheDOF4, PheDOF6 | (62) |
|  | LFY consensus binding motif | LFY | (147, 148) |
|  | GATA box | MNP | (149) |
| Anther specific expression and anther development | MYB recognition site | MYB26 | (150) |
|  | GTGA |  | (151) |
| Pollen specific expression and pollen development | MYB recognition site | MYB2 | (152) |
|  | POLLEN1LELAT52 | LAT52 | (153) |
|  | GTGA |  | (154-157) |

**References for supplemental table 2:**

1. G. Yi *et al.*, MYB1 transcription factor is a candidate responsible for red root skin in radish (Raphanus sativus L.). *PLOS ONE* **13**, e0204241 (2018).

2. K. Lin-Wang *et al.*, An R2R3 MYB transcription factor associated with regulation of the anthocyanin biosynthetic pathway in Rosaceae. *BMC plant biology* **10**, 50 (2010).

3. K. E. Schwinn *et al.*, The Onion (Allium cepa L.) R2R3-MYB Gene MYB1 Regulates Anthocyanin Biosynthesis. *Frontiers in Plant Science* **7** (2016).

4. M. Ramya *et al.*, MYB1 transcription factor regulation through floral scent in Cymbidium cultivar ‘Sael Bit’. *Phytochemistry Letters* **32**, 181-187 (2019).

5. C. Bomal *et al.*, Involvement of Pinus taeda MYB1 and MYB8 in phenylpropanoid metabolism and secondary cell wall biogenesis: a comparative in planta analysis. *Journal of Experimental Botany* **59**, 3925-3939 (2008).

6. J. H. Jun, C. Liu, X. Xiao, R. A. Dixon, The Transcriptional Repressor MYB2 Regulates Both Spatial and Temporal Patterns of Proanthocyandin and Anthocyanin Pigmentation in Medicago truncatula  *The Plant Cell* **27**, 2860-2879 (2015).

7. H. Jiang *et al.*, The transcription factor MdMYB2 influences cold tolerance and anthocyanin accumulation by activating SUMO E3 ligase MdSIZ1 in apple. *Plant Physiology* **189**, 2044-2060 (2022).

8. S. K. Mondal, S. Roy, Genome-wide sequential, evolutionary, organizational and expression analyses of phenylpropanoid biosynthesis associated MYB domain transcription factors in Arabidopsis. *Journal of Biomolecular Structure and Dynamics* **36**, 1577-1601 (2018).

9. M. Zhou *et al.*, LNK1 and LNK2 Corepressors Interact with the MYB3 Transcription Factor in Phenylpropanoid Biosynthesis. *Plant Physiology* **174**, 1348-1358 (2017).

10. M. Mitra, P. Agarwal, S. Roy, The N-terminal MYB domains affect the stability and folding aspects of Arabidopsis thaliana MYB4 transcription factor under thermal stress. *Protoplasma* **258**, 633-650 (2021).

11. Q. Luo *et al.*, Isolation and molecular characterization of NtMYB4a, a putative transcription activation factor involved in anthocyanin synthesis in tobacco. *Gene* **760**, 144990 (2020).

12. X.-C. Wang *et al.*, Arabidopsis MYB4 plays dual roles in flavonoid biosynthesis. *The Plant Journal* **101**, 637-652 (2020).

13. K. Odgerel *et al.*, Effects of the repression of GIGANTEA gene StGI.04 on the potato leaf transcriptome and the anthocyanin content of tuber skin. *BMC plant biology* **22**, 249 (2022).

14. C. Schluttenhofer, L. Yuan, Regulation of specialized metabolism by WRKY transcription factors. *Plant Physiology* **167**, 295-306 (2015).

15. S. Gupta *et al.*, Insights into structural and functional diversity of Dof (DNA binding with one finger) transcription factor. *Planta* **241**, 549-562 (2015).

16. G. Gualberti *et al.*, Mutations in the Dof Zinc Finger Genes DAG2 and DAG1 Influence with Opposite Effects the Germination of Arabidopsis Seeds. *The Plant Cell* **14**, 1253-1263 (2002).

17. M. Papi *et al.*, Inactivation of the Phloem-Specific Dof Zinc Finger GeneDAG1 Affects Response to Light and Integrity of the Testa of Arabidopsis Seeds. *Plant Physiology* **128**, 411-417 (2002).

18. D. H. Park *et al.*, The Arabidopsis COG1 gene encodes a Dof domain transcription factor and negatively regulates phytochrome signaling. *The Plant Journal* **34**, 161-171 (2003).

19. H.-G. Kang, R. C. Foley, L. Oñate-Sánchez, C. Lin, K. B. Singh, Target genes for OBP3, a Dof transcription factor, include novel basic helix-loop-helix domain proteins inducible by salicylic acid. *The Plant Journal* **35**, 362-372 (2003).

20. H.-G. Kang, K. B. Singh, Characterization of salicylic acid-responsive, Arabidopsis Dof domain proteins: overexpression of OBP3 leads to growth defects. *The Plant Journal* **21**, 329-339 (2000).

21. S. Yanagisawa, J. Sheen, Involvement of Maize Dof Zinc Finger Proteins in Tissue-Specific and Light-Regulated Gene Expression. *The Plant Cell* **10**, 75-89 (1998).

22. J. Yang, M.-F. Yang, D. Wang, F. Chen, S.-H. Shen, JcDof1, a Dof transcription factor gene, is associated with the light-mediated circadian clock in Jatropha curcas. *Physiologia Plantarum* **139**, 324-334 (2010).

23. W. B. Terzaghi, A. R. Cashmore, Light-Regulated Transcription. *Annual Review of Plant Physiology and Plant Molecular Biology* **46**, 445-474 (1995).

24. P. B. F. Ouwerkerk, T. O. Trimborn, F. Hilliou, J. Memelink, Nuclear factors GT-1 and 3AF1 interact with multiple sequences within the promoter of the Tdc gene from Madagascar periwinkle: GT-1 is involved in UV light-induced expression. *Molecular and General Genetics MGG* **261**, 610-622 (1999).

25. R. Wang, G. Hong, B. Han, Transcript abundance of rml1, encoding a putative GT1-like factor in rice, is up-regulated by Magnaporthe grisea and down-regulated by light. *Gene* **324**, 105-115 (2004).

26. E. Lam, N.-H. Chua, GT-1 binding site confers light responsive expression in transgenic tobacco. *Science* **248**, 471-474 (1990).

27. U. Borello, E. Ceccarelli, G. Giuliano, Constitutive, light-responsive and circadian clock-responsive factors compete for the different I box elements in plant light-regulated promoters. *The Plant Journal* **4**, 611-619 (1993).

28. R. G. Donald, A. R. Cashmore, Mutation of either G box or I box sequences profoundly affects expression from the Arabidopsis rbcS-1A promoter. *The EMBO Journal* **9**, 1717-1726 (1990).

29. K. Baum, B. Gröning, I. Meier, Improved ballistic transient transformation conditions for tomato fruit allow identification of organ-specific contributions of I-box and G-box to the RBCS2 promoter activity. *The Plant Journal* **12**, 463-469 (1997).

30. A. Rose, I. Meier, U. Wienand, The tomato I-box binding factor LeMYBI is a member of a novel class of Myb-like proteins. *The Plant Journal* **20**, 641-652 (1999).

31. M. Chakraborty *et al.*, Functional interrelation of MYC2 and HY5 plays an important role in Arabidopsis seedling development. *The Plant Journal* **99**, 1080-1097 (2019).

32. F. Rook, P. Weisbeek, S. Smeekens, The light-regulated Arabidopsis bZIP transcription factor gene ATB2 encodes a protein with an unusually long leucine zipper domain. *Plant Molecular Biology* **37**, 171-178 (1998).

33. K. Baek *et al.*, Introducing Dunaliella LIP promoter containing light‐inducible motifs improves transgenic expression in Chlamydomonas reinhardtii. *Biotechnology Journal* **11**, 384-392 (2016).

34. M. E. Hudson, P. H. Quail, Identification of promoter motifs involved in the network of phytochrome A-regulated gene expression by combined analysis of genomic sequence and microarray data. *Plant physiology* **133**, 1605-1616 (2003).

35. T. Kawoosa, P. Gahlan, A. S. Devi, S. Kumar, The GATA and SORLIP motifs in the 3-hydroxy-3-methylglutaryl-CoA reductase promoter of Picrorhiza kurrooa for the control of light-mediated expression. *Functional & integrative genomics* **14**, 191-203 (2014).

36. A. M. Rus Alvarez-Canterbury, D. J. Flores, K. Keymanesh, K. To, J. A. Brusslan, A double SORLIP1 element is required for high light induction of ELIP genes in Arabidopsis thaliana. *Plant molecular biology* **84**, 259-267 (2014).

37. G. R. Teakle, I. W. Manfield, J. F. Graham, P. M. Gilmartin, Arabidopsis thaliana GATA factors: organisation, expression and DNA-binding characteristics. *Plant molecular biology* **50**, 43-56 (2002).

38. I. W. Manfield, P. F. Devlin, C.-H. Jen, D. R. Westhead, P. M. Gilmartin, Conservation, convergence, and divergence of light-responsive, circadian-regulated, and tissue-specific expression patterns during evolution of the Arabidopsis GATA gene family. *Plant physiology* **143**, 941-958 (2007).

39. Q. L. Ranftl, E. Bastakis, C. Klermund, C. Schwechheimer, LLM-domain containing B-GATA factors control different aspects of cytokinin-regulated development in Arabidopsis thaliana. *Plant physiology* **170**, 2295-2311 (2016).

40. D. Hudson *et al.*, GNC and CGA1 modulate chlorophyll biosynthesis and glutamate synthase (GLU1/Fd-GOGAT) expression in Arabidopsis. *PloS one* **6**, e26765 (2011).

41. C. Yu *et al.*, Genome-wide identification and function characterization of GATA transcription factors during development and in response to abiotic stresses and hormone treatments in pepper. *Journal of Applied Genetics* **62**, 265-280 (2021).

42. C. Vannini *et al.*, Evaluation of transgenic tomato plants ectopically expressing the rice Osmyb4 gene. *Plant Science* **173**, 231-239 (2007).

43. M. N. Al-Attala, X. Wang, M. A. Abou-Attia, X. Duan, Z. Kang, A novel TaMYB4 transcription factor involved in the defence response against Puccinia striiformis f. sp. tritici and abiotic stresses. *Plant Molecular Biology* **84**, 589-603 (2014).

44. A. S. Buchel, R. Molenkamp, J. F. Bol, H. J. M. Linthorst, The PR-1a promoter contains a number of elements that bind GT-1-like nuclear factors with different affinity. *Plant Molecular Biology* **30**, 493-504 (1996).

45. H. C. Park *et al.*, Pathogen- and NaCl-Induced Expression of the SCaM-4 Promoter Is Mediated in Part by a GT-1 Box That Interacts with a GT-1-Like Transcription Factor. *Plant Physiology* **135**, 2150-2161 (2004).

46. H. Liu *et al.*, NBS-LRR protein Pik-H4 Interacts with OsBIHD1 to Balance Rice Blast Resistance and Growth by coordinating Ethylene-Brassinosteroid pathway. *Frontiers in Plant Science* **8** (2017).

47. H. Luo, F. Song, R. Goodman, Z. Zheng, Up-regulation of OsBIHD1, a rice gene encoding BELL homeodomain transcriptional factor, in disease resistance responses. *Plant Biology* **7**, 459-468 (2005).

48. H. Luo, F. Song, Z. Zheng, Overexpression in transgenic tobacco reveals different roles for the rice homeodomain gene OsBIHD1 in biotic and abiotic stress responses. *Journal of Experimental Botany* **56**, 2673-2682 (2005).

49. T. Eulgem, I. E. Somssich, Networks of WRKY transcription factors in defense signaling. *Current opinion in plant biology* **10**, 366-371 (2007).

50. M. Skibbe, N. Qu, I. Galis, I. T. Baldwin, Induced Plant Defenses in the Natural Environment: Nicotiana attenuata WRKY3 and WRKY6 Coordinate Responses to Herbivory. *The Plant Cell* **20**, 1984-2000 (2008).

51. R. Li *et al.*, Prioritizing plant defence over growth through WRKY regulation facilitates infestation by non-target herbivores. *eLife* **4**, e04805 (2015).

52. L. Hu, M. Ye, R. Li, Y. Lou, OsWRKY53, a versatile switch in regulating herbivore-induced defense responses in rice. *Plant Signaling & Behavior* **11**, e1169357 (2016).

53. P. Kundu, J. Vadassery, Role of WRKY transcription factors in plant defense against lepidopteran insect herbivores: an overview. *Journal of Plant Biochemistry and Biotechnology* **30**, 698-707 (2021).

54. N. Khurana, H. Chauhan, P. Khurana, Wheat Chloroplast Targeted sHSP26 Promoter Confers Heat and Abiotic Stress Inducible Expression in Transgenic Arabidopsis Plants. *PLOS ONE* **8**, e54418 (2013).

55. D. Kummari, P. Bhatnagar-Mathur, K. K. Sharma, V. Vadez, S. R. Palakolanu, Functional characterization of the promoter of pearl millet heat shock protein 10 (PgHsp10) in response to abiotic stresses in transgenic tobacco plants. *International Journal of Biological Macromolecules* **156**, 103-110 (2020).

56. M. Rieping, F. Schöffl, Synergistic effect of upstream sequences, CCAAT box elements, and HSE sequences for enhanced expression of chimaeric heat shock genes in transgenic tobacco. *Molecular and General Genetics MGG* **231**, 226-232 (1992).

57. Z. Zhang *et al.*, Opposing control by transcription factors MYB61 and MYB3 increases freezing tolerance by relieving C-repeat binding factor suppression. *Plant physiology* **172**, 1306-1323 (2016).

58. G. Pasquali, S. Biricolti, F. Locatelli, E. Baldoni, M. Mattana, Osmyb4 expression improves adaptive responses to drought and cold stress in transgenic apples. *Plant Cell Reports* **27**, 1677-1686 (2008).

59. C. Vannini *et al.*, Overexpression of the rice Osmyb4 gene increases chilling and freezing tolerance of Arabidopsis thaliana plants. *The Plant Journal* **37**, 115-127 (2004).

60. J. Jeon, J. Kim, Arabidopsis Response Regulator1 and Arabidopsis Histidine Phosphotransfer Protein2 (AHP2), AHP3, and AHP5 Function in Cold Signaling    *Plant Physiology* **161**, 408-424 (2013).

61. J. Zhu *et al.*, Low Temperature Inhibits Root Growth by Reducing Auxin Accumulation via ARR1/12. *Plant and Cell Physiology* **56**, 727-736 (2015).

62. Z. Cheng *et al.*, Characterization of moso bamboo (Phyllostachys edulis) Dof transcription factors in floral development and abiotic stress responses. *Genome* **61**, 151-156 (2018).

63. Y. Su *et al.*, Overexpression of GhDof1 improved salt and cold tolerance and seed oil content in Gossypium hirsutum. *Journal of Plant Physiology* **218**, 222-234 (2017).

64. C. Ren *et al.*, Characterization of Chromatin Accessibility and Gene Expression upon Cold Stress Reveals that the RAV1 Transcription Factor Functions in Cold Response in Vitis Amurensis. *Plant and Cell Physiology* **62**, 1615-1629 (2021).

65. V. Chinnusamy *et al.*, ICE1: a regulator of cold-induced transcriptome and freezing tolerance in Arabidopsis. *Genes & development* **17**, 1043-1054 (2003).

66. W. Xu *et al.*, Chinese Wild-Growing Vitis amurensis ICE1 and ICE2 Encode MYC-Type bHLH Transcription Activators that Regulate Cold Tolerance in Arabidopsis. *PLOS ONE* **9**, e102303 (2014).

67. X. Lu *et al.*, A novel Zea mays ssp. mexicana L. MYC-type ICE-like transcription factor gene ZmmICE1, enhances freezing tolerance in transgenic Arabidopsis thaliana. *Plant Physiology and Biochemistry* **113**, 78-88 (2017).

68. Z.-F. Zuo *et al.*, Zoysia japonica MYC type transcription factor ZjICE1 regulates cold tolerance in transgenic Arabidopsis. *Plant Science* **289**, 110254 (2019).

69. J. B. Song *et al.*, miR394 and its target gene LCR are involved in cold stress response in Arabidopsis. *Plant Gene* **5**, 56-64 (2016).

70. L. Xiang *et al.*, The cold-induced transcription factor bHLH112 promotes artemisinin biosynthesis indirectly via ERF1 in Artemisia annua. *Journal of Experimental Botany* **70**, 4835-4848 (2019).

71. W. Li, S. Pang, Z. Lu, B. Jin, Function and Mechanism of WRKY Transcription Factors in Abiotic Stress Responses of Plants. *Plants* **9**, 1515 (2020).

72. J. A. Massange-Sánchez *et al.*, Overexpression of Grain Amaranth (Amaranthus hypochondriacus) AhERF or AhDOF Transcription Factors in Arabidopsis thaliana Increases Water Deficit- and Salt-Stress Tolerance, Respectively, via Contrasting Stress-Amelioration Mechanisms. *PLOS ONE* **11**, e0164280 (2016).

73. A.-R. Corrales *et al.*, Characterization of tomato Cycling Dof Factors reveals conserved and new functions in the control of flowering time and abiotic stress responses. *Journal of Experimental Botany* **65**, 995-1012 (2014).

74. C. Li *et al.*, The bHLH transcription factor AhbHLH112 improves the drought tolerance of peanut. *BMC plant biology* **21**, 540 (2021).

75. Y. Liu *et al.*, Arabidopsis AtbHLH112 regulates the expression of genes involved in abiotic stress tolerance by binding to their E-box and GCG-box motifs. *New Phytologist* **207**, 692-709 (2015).

76. S. Sengupta, A. Ray, D. Mandal, R. Nag Chaudhuri, ABI3 mediated repression of RAV1 gene expression promotes efficient dehydration stress response in Arabidopsis thaliana. *Biochimica et Biophysica Acta (BBA) - Gene Regulatory Mechanisms* **1863**, 194582 (2020).

77. H. Abe *et al.*, Arabidopsis AtMYC2 (bHLH) and AtMYB2 (MYB) function as transcriptional activators in abscisic acid signaling. *The Plant Cell* **15**, 63-78 (2003).

78. H. Abe *et al.*, Role of arabidopsis MYC and MYB homologs in drought-and abscisic acid-regulated gene expression. *The Plant Cell* **9**, 1859-1868 (1997).

79. J. B. Song *et al.*, miR394 and LCR are involved in Arabidopsis salt and drought stress responses in an abscisic acid-dependent manner. *BMC plant biology* **13**, 210 (2013).

80. X. Ji *et al.*, The bZIP protein from Tamarix hispida, ThbZIP1, is ACGT elements binding factor that enhances abiotic stress signaling in transgenic Arabidopsis. *BMC plant biology* **13**, 151 (2013).

81. R. Mehrotra, S. Mehrotra, Promoter activation by ACGT in response to salicylic and abscisic acids is differentially regulated by the spacing between two copies of the motif. *Journal of Plant physiology* **167**, 1214-1218 (2010).

82. J. H. Yoo *et al.*, Direct Interaction of a Divergent CaM Isoform and the Transcription Factor, MYB2, Enhances Salt Tolerance in <em>Arabidopsis</em>*. *Journal of Biological Chemistry* **280**, 3697-3706 (2005).

83. T. Urao, K. Yamaguchi-Shinozaki, S. Urao, K. Shinozaki, An Arabidopsis myb homolog is induced by dehydration stress and its gene product binds to the conserved MYB recognition sequence. *The Plant Cell* **5**, 1529-1539 (1993).

84. D. Zang, L. Wang, Y. Zhang, H. Zhao, Y. Wang, ThDof1.4 and ThZFP1 constitute a transcriptional regulatory cascade involved in salt or osmotic stress in Tamarix hispida. *Plant Molecular Biology* **94**, 495-507 (2017).

85. X. Cai *et al.*, The transcription factor SlDof22 involved in ascorbate accumulation and salinity stress in tomato. *Biochemical and Biophysical Research Communications* **474**, 736-741 (2016).

86. P. Hu, K. Zhang, C. Yang, Functional roles of the birch BpRAV1 transcription factor in salt and osmotic stress response. *Plant Science* **315**, 111131 (2022).

87. H. Min, J. Zheng, J. Wang, Maize ZmRAV1 contributes to salt and osmotic stress tolerance in transgenic arabidopsis. *Journal of Plant Biology* **57**, 28-42 (2014).

88. K. H. Sohn, S. C. Lee, H. W. Jung, J. K. Hong, B. K. Hwang, Expression and functional roles of the pepper pathogen-induced transcription factor RAV1 in bacterial disease resistance, and drought and salt stress tolerance. *Plant molecular biology* **61**, 897-915 (2006).

89. R. Mehrotra, S. Sethi, I. Zutshi, P. Bhalothia, S. Mehrotra, Patterns and evolution of ACGT repeat cis-element landscape across four plant genomes. *BMC Genomics* **14**, 203 (2013).

90. R. Satoh, Y. Fujita, K. Nakashima, K. Shinozaki, K. Yamaguchi-Shinozaki, A Novel Subgroup of bZIP Proteins Functions as Transcriptional Activators in Hypoosmolarity-Responsive Expression of the ProDH Gene in Arabidopsis. *Plant and Cell Physiology* **45**, 309-317 (2004).

91. T. G. Lee *et al.*, A Myb transcription factor (TaMyb1) from wheat roots is expressed during hypoxia: roles in response to the oxygen concentration in root environment and abiotic stresses. *Physiologia Plantarum* **129**, 375-385 (2007).

92. F. U. Hoeren, R. Dolferus, Y. Wu, W. J. Peacock, E. S. Dennis, Evidence for a role for AtMYB2 in the induction of the Arabidopsis alcohol dehydrogenase gene (ADH1) by low oxygen. *Genetics* **149**, 479-490 (1998).

93. S. Zhu, W. Shi, Y. Jie, Q. Zhou, C. Song, A MYB transcription factor, BnMYB2, cloned from ramie (Boehmeria nivea) is involved in cadmium tolerance and accumulation. *PLOS ONE* **15**, e0233375 (2020).

94. P. Agarwal, M. Mitra, S. Banerjee, S. Roy, MYB4 transcription factor, a member of R2R3-subfamily of MYB domain protein, regulates cadmium tolerance via enhanced protection against oxidative damage and increases expression of PCS1 and MT1C in Arabidopsis. *Plant Science* **297**, 110501 (2020).

95. G. N. Raldugina *et al.*, Expression of rice OsMyb4 transcription factor improves tolerance to copper or zinc in canola plants. *Biologia Plantarum* **62**, 511-520 (2018).

96. Y. Yang *et al.*, Transcriptome-wide analysis of jasmonate-treated BY-2 cells reveals new transcriptional regulators associated with alkaloid formation in tobacco. *Journal of Plant Physiology* **215**, 1-10 (2017).

97. M. Boter, O. Ruíz-Rivero, A. Abdeen, S. Prat, Conserved MYC transcription factors play a key role in jasmonate signaling both in tomato and Arabidopsis. *Genes & development* **18**, 1577-1591 (2004).

98. O. Lorenzo, J. M. Chico, J. J. Saénchez-Serrano, R. Solano, JASMONATE-INSENSITIVE1 encodes a MYC transcription factor essential to discriminate between different jasmonate-regulated defense responses in Arabidopsis. *The Plant Cell* **16**, 1938-1950 (2004).

99. P. Figueroa, J. Browse, The Arabidopsis JAZ2 Promoter Contains a G-Box and Thymidine-Rich Module that are Necessary and Sufficient for Jasmonate-Dependent Activation by MYC Transcription Factors and Repression by JAZ Proteins. *Plant and Cell Physiology* **53**, 330-343 (2011).

100. A. Chini, S. Fonseca, J. M. Chico, P. Fernández‐Calvo, R. Solano, The ZIM domain mediates homo‐and heteromeric interactions between Arabidopsis JAZ proteins. *The Plant Journal* **59**, 77-87 (2009).

101. H. S. Chung, G. A. Howe, A critical role for the TIFY motif in repression of jasmonate signaling by a stabilized splice variant of the JASMONATE ZIM-domain protein JAZ10 in Arabidopsis. *The Plant Cell* **21**, 131-145 (2009).

102. Y. Guo, S. Gan, AtMYB2 regulates whole plant senescence by inhibiting cytokinin-mediated branching at late stages of development in Arabidopsis. *Plant physiology* **156**, 1612-1619 (2011).

103. H. Sakai *et al.*, ARR1, a transcription factor for genes immediately responsive to cytokinins. *Science* **294**, 1519-1521 (2001).

104. E. J. H. Ross *et al.*, Activation of the Oryza sativa non-symbiotic haemoglobin-2 promoter by the cytokinin-regulated transcription factor, ARR1. *Journal of experimental botany* **55**, 1721-1731 (2004).

105. M. G. Mason *et al.*, Type-B response regulators ARR1 and ARR12 regulate expression of AtHKT1;1 and accumulation of sodium in Arabidopsis shoots. *The Plant Journal* **64**, 753-763 (2010).

106. A. Cortleven *et al.*, A Novel Protective Function for Cytokinin in the Light Stress Response Is Mediated by the ARABIDOPSIS HISTIDINE KINASE2 and ARABIDOPSIS HISTIDINE KINASE3 Receptors  *Plant Physiology* **164**, 1470-1483 (2014).

107. D. Mandal, S. Datta, G. Ravindra, P. K. Mondal, R. N. Chaudhuri, RAV1 mediates cytokinin signalling for regulating primary root growth in Arabidopsis. *bioRxiv* 10.1101/2022.07.13.499994, 2022.2007.2013.499994 (2022).

108. J. Dong, C. Chen, Z. Chen, Expression profiles of the Arabidopsis WRKY gene superfamily during plant defense response. *Plant molecular biology* **51**, 21-37 (2003).

109. D. Yu, C. Chen, Z. Chen, Evidence for an important role of WRKY DNA binding proteins in the regulation of NPR1 gene expression. *The Plant Cell* **13**, 1527-1540 (2001).

110. H. Chen *et al.*, AcoMYB4, an Ananas comosus L. MYB Transcription Factor, Functions in Osmotic Stress through Negative Regulation of ABA Signaling. <http://dx.doi.org/10.3390/ijms21165727>.

111. S. Y. Kim, H. J. Chung, T. L. Thomas, Isolation of a novel class of bZIP transcription factors that interact with ABA‐responsive and embryo‐specification elements in the Dc3 promoter using a modified yeast one‐hybrid system. *The Plant Journal* **11**, 1237-1251 (1997).

112. S. Y. Kim, T. L. Thomas, A family of novel basic leucine zipper proteins binds to seed-specification elements in the carrot Dc3 gene promoter. *Journal of plant physiology* **152**, 607-613 (1998).

113. S. Y. Kim, J. Ma, P. Perret, Z. Li, T. L. Thomas, Arabidopsis ABI5 Subfamily Members Have Distinct DNA-Binding and Transcriptional Activities. *Plant Physiology* **130**, 688-697 (2002).

114. R. R. Finkelstein, T. J. Lynch, The Arabidopsis Abscisic Acid Response Gene ABI5 Encodes a Basic Leucine Zipper Transcription Factor. *The Plant Cell* **12**, 599-609 (2000).

115. Y. Huang, C.-Z. Feng, Q. Ye, W.-H. Wu, Y.-F. Chen, Arabidopsis WRKY6 Transcription Factor Acts as a Positive Regulator of Abscisic Acid Signaling during Seed Germination and Early Seedling Development. *PLOS Genetics* **12**, e1005833 (2016).

116. C. Z. Feng *et al.*, Arabidopsis RAV 1 transcription factor, phosphorylated by S n RK 2 kinases, regulates the expression of ABI 3, ABI 4, and ABI 5 during seed germination and early seedling development. *The Plant Journal* **80**, 654-668 (2014).

117. M. a. Mena, F. J. Cejudo, I. Isabel-Lamoneda, P. Carbonero, A Role for the DOF Transcription Factor BPBF in the Regulation of Gibberellin-Responsive Genes in Barley Aleurone. *Plant Physiology* **130**, 111-119 (2002).

118. R. Richter, C. Behringer, I. K. Müller, C. Schwechheimer, The GATA-type transcription factors GNC and GNL/CGA1 repress gibberellin signaling downstream from DELLA proteins and PHYTOCHROME-INTERACTING FACTORS. *Genes & Development* **24**, 2093-2104 (2010).

119. X.-M. Luo *et al.*, Integration of light-and brassinosteroid-signaling pathways by a GATA transcription factor in Arabidopsis. *Developmental cell* **19**, 872-883 (2010).

120. J. Chen *et al.*, Arabidopsis WRKY46, WRKY54, and WRKY70 Transcription Factors Are Involved in Brassinosteroid-Regulated Plant Growth and Drought Responses. *The Plant Cell* **29**, 1425-1439 (2017).

121. J. Chen, Y. Yin, WRKY transcription factors are involved in brassinosteroid signaling and mediate the crosstalk between plant growth and drought tolerance. *Plant Signaling & Behavior* **12**, e1365212 (2017).

122. Z. Wei *et al.*, Brassinosteroid Biosynthesis Is Modulated via a Transcription Factor Cascade of COG1, PIF4, and PIF5. *Plant Physiology* **174**, 1260-1273 (2017).

123. Y. Zhang, X. Ji, J. Xian, Y. Wang, Y. Peng, Morphological characterization and transcriptome analysis of leaf angle mutant bhlh112 in maize [Zea mays L.]. *Frontiers in Plant Science* **13** (2022).

124. Y. X. Hu, Y. H. Wang, X. F. Liu, J. Y. Li, Arabidopsis RAV1 is down-regulated by brassinosteroid and may act as a negative regulator during plant development. *Cell Research* **14**, 8-15 (2004).

125. I. Diaz *et al.*, The GAMYB protein from barley interacts with the DOF transcription factor BPBF and activates endosperm-specific genes during seed development. *The Plant Journal* **29**, 453-464 (2002).

126. R. D. Allen, F. Bernier, P. A. Lessard, R. N. Beachy, Nuclear factors interact with a soybean beta-conglycinin enhancer. *The Plant Cell* **1**, 623-631 (1989).

127. P. A. Lessard *et al.*, Multiple nuclear factors interact with upstream sequences of differentially regulated β-conglycinin genes. *Plant Molecular Biology* **16**, 397-413 (1991).

128. H. Y. Shin, K. H. Nam, RAV1 Negatively Regulates Seed Development by Directly Repressing MINI3 and IKU2 in Arabidopsis. *Mol Cells* **41**, 1072-1080 (2018).

129. P. P. Liu, N. Koizuka, R. C. Martin, H. Nonogaki, The BME3 (Blue Micropylar End 3) GATA zinc finger transcription factor is a positive regulator of Arabidopsis seed germination. *The Plant Journal* **44**, 960-971 (2005).

130. C.-Y. Wu, H. Washida, Y. Onodera, K. Harada, F. Takaiwa, Quantitative nature of the Prolamin-box, ACGT and AACA motifs in a rice glutelin gene promoter: minimal cis-element requirements for endosperm-specific gene expression. *The Plant Journal* **23**, 415-421 (2000).

131. H. R. Woo *et al.*, The RAV1 transcription factor positively regulates leaf senescence in Arabidopsis. *Journal of Experimental Botany* **61**, 3947-3957 (2010).

132. U. Gowik *et al.*, cis-Regulatory elements for mesophyll-specific gene expression in the C4 plant Flaveria trinervia, the promoter of the C4 phosphoenolpyruvate carboxylase gene. *The Plant Cell* **16**, 1077-1090 (2004).

133. X. Han *et al.*, Jasmonate negatively regulates stomatal development in Arabidopsis cotyledons. *Plant Physiology* **176**, 2871-2885 (2018).

134. Y.-H. Chiang *et al.*, Functional characterization of the GATA transcription factors GNC and CGA1 reveals their key role in chloroplast development, growth, and division in Arabidopsis. *Plant physiology* **160**, 332-348 (2012).

135. Y. M. Bi *et al.*, Genetic analysis of Arabidopsis GATA transcription factor gene family reveals a nitrate‐inducible member important for chlorophyll synthesis and glucose sensitivity. *The Plant Journal* **44**, 680-692 (2005).

136. W.-S. Wang, J. Zhu, Y.-T. Lu, Overexpression of AtbHLH112 suppresses lateral root emergence in Arabidopsis. *Functional Plant Biology* **41**, 342-352 (2013).

137. T. Elmayan, M. Tepfer, Evaluation in tobacco of the organ specificity and strength of therolD promoter, domain A of the 35S promoter and the 35S2 promoter. *Transgenic Research* **4**, 388-396 (1995).

138. Y.-Q. Wang, S.-Y. Zhu, Y. Wang, M.-Y. Zhang, Tissue and inducible expression of a rice glutathione transporter gene promoter in transgenic Arabidopsis. *Bot Stud* **48**, 35-41 (2007).

139. V. Fehlberg *et al.*, The promoter of the leghaemoglobin gene VfLb29: functional analysis and identification of modules necessary for its activation in the infected cells of root nodules and in the arbuscule-containing cells of mycorrhizal roots. *Journal of Experimental Botany* **56**, 799-806 (2005).

140. B. De Rybel *et al.*, A novel aux/IAA28 signaling cascade activates GATA23-dependent specification of lateral root founder cell identity. *Current Biology* **20**, 1697-1706 (2010).

141. Y. Yu *et al.*, The BpMYB4 Transcription Factor From Betula platyphylla Contributes Toward Abiotic Stress Resistance and Secondary Cell Wall Biosynthesis. *Frontiers in Plant Science* **11** (2021).

142. M. Konishi, S. Yanagisawa, Sequential activation of two Dof transcription factor gene promoters during vascular development in Arabidopsis thaliana. *Plant Physiology and Biochemistry* **45**, 623-629 (2007).

143. H. Endo *et al.*, Multiple classes of transcription factors regulate the expression of VASCULAR-RELATED NAC-DOMAIN7, a master switch of xylem vessel differentiation. *Plant and Cell Physiology* **56**, 242-254 (2015).

144. J. P. Maurya, V. Sethi, S. N. Gangappa, N. Gupta, S. Chattopadhyay, Interaction of MYC2 and GBF1 results in functional antagonism in blue light-mediated Arabidopsis seedling development. *The Plant Journal* **83**, 439-450 (2015).

145. C. Behringer, C. Schwechheimer, B-GATA transcription factors–insights into their structure, regulation, and role in plant development. *Frontiers in plant science* **6**, 90 (2015).

146. M. Shikata *et al.*, Characterization of Arabidopsis ZIM, a member of a novel plant‐specific GATA factor gene family. *Journal of experimental botany* **55**, 631-639 (2004).

147. E. A. Schultz, G. W. Haughn, LEAFY, a Homeotic Gene That Regulates Inflorescence Development in Arabidopsis. *The Plant Cell* **3**, 771-781 (1991).

148. L.-L. Wang, H.-M. Liang, J.-L. Pang, M.-Y. Zhu, Regulation network and biological roles of LEAFY in Arabidopsis thaliana in floral development. *Yi Chuan= Hereditas* **26**, 137-142 (2004).

149. Y. Zhao *et al.*, HANABA TARANU is a GATA transcription factor that regulates shoot apical meristem and flower development in Arabidopsis. *The Plant Cell* **16**, 2586-2600 (2004).

150. S. Steiner‐Lange *et al.*, Disruption of Arabidopsis thaliana MYB26 results in male sterility due to non‐dehiscent anthers. *The Plant Journal* **34**, 519-528 (2003).

151. X. Liu, Y. Shangguan, J. Zhu, Y. Lu, B. Han, The rice OsLTP6 gene promoter directs anther-specific expression by a combination of positive and negative regulatory elements. *Planta* **238**, 845-857 (2013).

152. X. Guo *et al.*, MYB2 Is Important for Tapetal PCD and Pollen Development by Directly Activating Protease Expression in Arabidopsis. <http://dx.doi.org/10.3390/ijms23073563>.

153. N. Bate, D. Twell, Functional architecture of a late pollen promoter: pollen-specific transcription is developmentally regulated by multiple stage-specific and co-dependent activator elements. *Plant molecular biology* **37**, 859-869 (1998).

154. H. J. Rogers *et al.*, Functional analysis of cis-regulatory elements within the promoter of the tobacco late pollen gene g10. *Plant Molecular Biology* **45**, 577-585 (2001).

155. S. Yan *et al.*, Functional architecture of two exclusively late stage pollen-specific promoters in rice (Oryza sativa L.). *Plant Molecular Biology* **88**, 415-428 (2015).

156. P. Manimaran *et al.*, Identification of cis-elements and evaluation of upstream regulatory region of a rice anther-specific gene, OSIPP3, conferring pollen-specific expression in Oryza sativa (L.) ssp. indica. *Plant Reproduction* **28**, 133-142 (2015).

157. H. Wang *et al.*, Isolation and characterization of a novel pollen-specific promoter in maize (Zea mays L.). *Genome* **60**, 485-495 (2017).

**Table S3: CREs moderately overrepresented in promoters of isoprene responsive genes.** 50-75% of the promoters were enriched in that motif for upregulated genes or downregulated genes or both groups. UR: underrepresented

| **CRE** | **Interacting TF or protein** | **% of upregulated genes with CRE** | **% of down-regulated genes with CRE** |
| --- | --- | --- | --- |
| ABRE-like-binding site motif | TRAB1, ABFs | 74 | UR |
| REα | Phytochrome | 72 |  |
| CuRE | CRR1 SPL gene family | 70 |  |
| Pyrimidine box | BPBF | 69 |  |
| GARE (GA- responsive element) | YAB1 | 67 |  |
| SURE | ARFs SLIM1 | 67 |  |
| PREAT | ATB2 | 67 |  |
| CArG motif | AGL15 MADS TF Family | 66 | UR |
| MARTBOX | - | 64 |  |
| ARF element | ARFs | 64 | 69 |
| -10 PEHVPSBD |  | 59 |  |
| AMYBOX1 |  | 57 |  |
| HD-ZIP binding  cis-elements (L1 box) | GL2, ATML1, PDF2, HDG11, ATHB17, HD-ZIP IV TF | 57 | 65 |
| LTRE promoter motif | AP2/ERF | 57 | UR |
| CPB-BINDING motif | CPB | 56 |  |
| BBF1-binding motif | BBF1 | 56 |  |
| TATCCAY motif | MYBS1, S2, S3 | 52 |  |
| ASF-1 binding site | ASF-1, TGA-1, TGA-6 | 51 |  |
| Box II promoter motif | CPRF-1, -2, -3 | UR | 71 |
| CCA1 binding site motif | MYB-related TF | UR | 57 |

**Table S4:** **CREs underrepresented in promoters of isoprene responsive genes.** ≤49% of promoters were enriched in that motif for upregulated genes or downregulated genes or both groups. MO: moderately overrepresented

| **CRE** | **Interacting TF or protein** | **% of upregulated genes with CRE** | **% of down-regulated genes with CRE** |
| --- | --- | --- | --- |
| CIACADIANLELHC |  | 48 |  |
| Box II promoter motif |  | 46 |  |
| PRECONSCRHSP70A |  | 46 |  |
| SREATMSD |  | 44 |  |
| RHERPATEXPA7 |  | 43 |  |
| ERELEE4 | ERFs | 41 |  |
| CAREOSREP1 |  | 39 |  |
| S1FBOXSORPS1L21 |  | 39 |  |
| AACACOREOSGLUB AACA motif | MYB5 | 38 |  |
| ABRE-like binding site motif | TRAB1, ABFs | MO | 37 |
| CBF binding site motif | AP2-EREBP, CBF1, CBF2 | 36 | 2 |
| CCA1ATLHCB1 | CCA1 | 36 |  |
| CDA1ATCAB2 |  | 36 |  |
| ATHB-binding site motif | Homeobox TF family ATHB1, ATHB2, ATHB5, ATHB6 | 36 | 22 |
| RY-repeat promoter motif |  | 34 | 8 |
| BOX L PALBOXLPC |  | 31 |  |
| SP8BFIBSP8BIB SP8BFIBSP8AIB | Binding site of SPF1; SPF1 also binds to the SP8b; | 31 |  |
| CGACGOSAMY3 CGACG element |  | 30 |  |
| SITEIIATCYTC |  | 30 |  |
| CANBNNAPA (CA)n element |  | 28 |  |
| E2F binding site motif | E2F-DP | 28 |  |
| G-box promoter motif [LRE] |  | 28 | 33 |
| LECPLEACS2 |  | 28 |  |
| SEBFCONSSTPR10A | SEBF | 28 |  |
| T/G-box |  | 26 |  |
| CATATGGMSAUR (GGTCCCATGMSAUR) |  | 25 |  |
| CGCGBOXAT | AtSR1-6 | 23 |  |
| SV40COREENHAN |  | 23 |  |
| Evening Element promoter motif |  | 20 | 33 |
| Hexamer promoter motif |  | 20 | 22 |
| WUSATA | WUS | 20 |  |
| CArG motif | AGL15 MADS TF Family | MO | 20 |
| CEREGLUBOX3PSLEGA |  | 18 |  |
| NAPINMOTIFBN |  | 18 |  |
| QELEMENTZMZM13 |  | 18 |  |
| RBCSCONSENSUS |  | 18 |  |
| REBETALGLHCB21 |  | 18 |  |
| HSE | HSF | 16 | 10 |
| MARABOX1 |  | 16 |  |
| UP1ATMSD Up1" motif UP2ATMSD | GGCCCAWWW AAACCCTA | 15 |  |
| GCC-box promoter motif (AGCBOXNPGLB) Ethylene-Response Element (ERE) | ERFs | 13 |  |
| P1BS |  | 13 |  |
| BOXCPSAS1 | A light activated transcriptional repressor | 10 |  |
| BOX P (PALBOXPPC) | YTYYMMCMAMCMMC | 10 |  |
| 2SSEEDPROTBANAPA |  | 10 |  |
| GCN4OSGLUB1 | bZIP TFs that belong to maize Opaque-2-like proteins. | 10 |  |
| IRO2OS |  | 10 |  |
| Prolamine box PROLAMINBOXOSGLUB1 | PBF-1 (TF) | 10 |  |
| Box A (PALBOXAPC) |  | 8 |  |
| EMHVCHORD |  | 8 |  |
| TGTCACACMCUCUMISIN |  | 8 |  |
| TELO-box promoter motif |  | 8 | 6 |
| XYLAT |  | 8 |  |
| LTRE promoter motif | AP2/ERF | MO | 8 |
| AG binding site in AP3 | MADS | 7 | 2 |
| AMMORESIIUDCRNIA1 |  | 7 |  |
| ARS element (MARARS) |  | 7 |  |
| QARBNEXTA |  | 7 |  |
| SURE1STPAT21 |  | 7 |  |
| TGA1 binding site motif (HEXAT) | bZIP class of DNA binding proteins TGA1, GBF1 | 7 | 2 |
| AGMOTIFNTMYB2 | MYB2, AGP1 (GATA-type zinc finger protein) | 5 |  |
| BP5OSWX |  | 5 |  |
| CMSRE1IBSPOA |  | 5 |  |
| NONAMERMOTIFTAH3H4 |  | 5 |  |
| NRRBNEXTA |  | 5 |  |
| TCA1MOTIF |  | 5 |  |
| ABFs binding site motif | bZIP | 3 | 2 |
| AP1 binding site in AP3 | MADS | 3 |  |
| CACGCAATGMGH3 |  | 3 | 2 |
| CRTDREHVCBF2 CRT/DRE motif |  | 3 |  |
| GADOWNAT |  | 3 |  |
| HBOXCONSENSUSPVCHS |  | 3 |  |
| HEXMOTIFTAH3H4 |  | 3 |  |
| SBP-box promoter motif |  | 3 | 8 |
| WRECSAA01 |  | 3 |  |
| Z-BOX ZDNAFORMINGATCAB1 |  | 3 | 6 |
| 52/56 box |  | 3 |  |
| ARE1 |  | 2 |  |
| BS1EGCCR |  | 2 |  |
| CELLCYCLESC cell cycle box |  | 2 |  |
| CONSERVED11NTZMATP1 |  | 2 |  |
| D1GMAUX28 |  | 2 |  |
| D3GMAUX28 |  | 2 |  |
| ELRENTCHN50 |  | 2 |  |
| GMHDLGMVSPB |  | 2 |  |
| MNF1ZMPPC1 |  | 2 |  |
| Motif I |  | 2 |  |
| Octamer promoter motif |  | 2 |  |
| OPAQUE2ZMB32 |  | 2 |  |
| PI promoter motif |  | 2 | 2 |
| PRHA BS in PAL1 | HB | 2 |  |
| pE2F (proximal E2F element |  | 2 |  |
| PROXBBNNAPA |  | 2 |  |
| SGBFGMGMAUX28 | GBF-1 and SGBF-2 | 2 |  |
| SBOXATRBCS |  | 2 |  |
| UPRMOTIFIIAT |  | 2 |  |
| AGL3 | MADS | NF | 2 |
| LS7 |  | NF | 2 |

**Fig. S1:** Probability density of membrane components and isoprene for thylakoid membranes that contain (left) 0-mol% and (right) 20-mol% isoprene.
